# The effect of adverse childhood experience training, screening, and response in primary care: a systematic review

**DOI:** 10.1016/j.eclinm.2023.102282

**Published:** 2023-10-24

**Authors:** Ryan K. McBain, Jonathan S. Levin, Samantha Matthews, Nabeel Qureshi, Dayna Long, Adam B. Schickedanz, Rachel Gilgoff, Krista Kotz, George M. Slavich, Nicole K. Eberhart

**Affiliations:** a Department of Medicine, Brigham & Women's Hospital, Boston, MA, USA; b Division of Healthcare Delivery, RAND Corporation, Washington, DC, USA; c Division of Healthcare Delivery, RAND Corporation, Santa Monica, CA, USA; d Department of Medicine, University of California, San Francisco, CA, USA; e Department of Pediatrics, David Geffen School of Medicine and Department of Health Policy and Management, Fielding School of Public Health, University of California, Los Angeles, CA, USA; f UCLA-UCSF ACEs Aware Family Resilience Network, Los Angeles, CA, USA; g Department of Psychiatry and Biobehavioral Sciences, University of California, Los Angeles, CA, USA

**Keywords:** Traumatic stress, Adverse childhood experiences, Pediatrics, Primary care, Life stressors

## Abstract

**Background:**

Adverse childhood experiences (ACEs) can have harmful, long-term health effects. Although primary care providers (PCPs) could help mitigate these effects, no studies have reviewed the impacts of ACE training, screening, and response in primary care.

**Methods:**

This systematic review searched four electronic databases (PubMed, Web of Science, APA PsycInfo, CINAHL) for peer-reviewed articles on ACE training, screening, and/or response in primary care published between Jan 1, 1998, and May 31, 2023. Searches were limited to primary research articles in the primary care setting that reported provider-related outcomes (knowledge, confidence, screening behavior, clinical care) and/or patient-related outcomes (satisfaction, referral engagement, health outcomes). Summary data were extracted from published reports.

**Findings:**

Of 6532 records, 58 met inclusion criteria. Fifty-two reported provider-related outcomes; 21 reported patient-related outcomes. 50 included pediatric populations, 12 included adults. A majority discussed screening interventions (n = 40). Equal numbers (n = 25) discussed training and clinical response interventions. Strength of evidence (SOE) was generally low, especially for adult studies. This was due to reliance on observational evidence, small samples, and self-report measures for heterogeneous outcomes. Exceptions with moderate SOE included the effect of training interventions on provider confidence/self-efficacy and the effect of screening interventions on screening uptake and patient satisfaction.

**Interpretation:**

Primary care represents a potentially strategic setting for addressing ACEs, but evidence on patient- and provider-related outcomes remains scarce.

**Funding:**

The California Department of Health Care Services and the Office of the California Surgeon General.

**Research in context**

**Evidence before this study**

In a preliminary search of PubMed, Web of Science database, APA PsycInfo, and CINAHL, we assessed existing evidence on training, screening, and response to addressing adverse childhood experiences (ACEs) in primary care between January 1998 and May 2023, not restricted to the English language. Our search terms included primary care setting terminology (“primary care”, “pediatrics”) separated by OR statements, conjoined using AND statements with ACE-related terminology (“adverse childhood experience”, “childhood adversity”) also separated by OR statements. We identified a number of studies on scale-up of screening for ACEs in primary care settings, as well as epidemiologic studies on ACE prevalence and demographic differences in these settings. A 2022 systematic review on patient-to-provider communication of childhood adversity in primary care identified five studies from 2011 through 2021 and concluded that effective communication is essential for addressing ACE exposure; however, it did not link implementation of ACE screening in primary care with patient or provider outcomes.

**Added value of this study**

This study uses a comprehensive search strategy to synthesize evidence on the effects of ACE training, screening, and clinical response in primary care—from the vantage point of provider- and patient-related outcomes. We found 58 studies that met inclusion criteria, collectively providing information of the state of the science on addressing ACEs in primary care settings. We found that the current strength of evidence to support interventions is low or very low with respect to most outcomes, including enhanced provider knowledge, changes in clinical behavior, improved patient-provider rapport, and altered health outcomes. Evidence was strongest (moderate) for intervention effects on provider confidence when discussing composite ACEs and patient acceptability and satisfaction with care. We did not find any evidence of iatrogenic effects.

**Implications of all the available evidence**

This systematic review demonstrates that there is a dearth of evidence linking screening for ACEs and clinical response interventions in primary care settings with respect to patient and provider outcomes. We found few studies outside high-income countries, despite the vast majority of children and adolescents living in low- and middle-income countries. Furthermore, only 12 studies evaluated ACE-related assessments of adults, compared to 50 studies that included pediatric populations. Given the rapid and widespread scale-up of ACE screening and response in primary care settings, this study provides impetus for more rigorous evaluations. Although the limited evidence to-date suggests addressing ACEs in primary care may yield benefits, more rigorous investigations would help clinicians discern which types of interventions are most effective in addressing ACEs outside specialty care settings.

## Introduction

Adverse childhood experiences (ACEs) represent a constellation of potentially traumatic life stressors that occur during childhood and adolescence.^1^ In a seminal CDC-Kaiser Permanente study and follow-on research,^2^^,^^3^ a focused set of ACEs were identified—including forms of child abuse and neglect, parental intimate partner violence and divorce, and incarceration and behavioral health problems among household members. Unaddressed, ACEs can contribute to the activation and dysregulation of physiologic stress-response systems, which can alter life-long health and development.^4^ This includes serious health problems such as cardiovascular disease, diabetes, cancers, and mental health conditions.^5^ Individuals with six or more ACEs have a life expectancy that is, on average, 20 years shorter compared to those with no ACEs.^6^

A growing literature has delineated the potential utility of screening for and providing services that countervail the effects of varying ACE exposures. For example, a recent review classified seven types of interventions shown to have varying levels of benefits for those with a history of ACEs.^7^ Likewise, curricula have been developed to educate health care providers on best practices for ACE training, screening, and clinical response.^8^^,^^9^ This includes application of validated, composite ACE screening instruments, such as the original CDC-Kaiser Permanente Questionnaire, and the use of evidence-based practices, such as trauma-informed care, in response to the identification of ACEs.^10^^,^^11^

Primary care settings are meant to be routine entry points into the health system, where primary care providers (PCPs) can gradually cultivate rapport and trust with their patients.^12^ PCPs administer screenings and evaluations to assess overall health and wellbeing, creating an opportunity to incorporate ACE screening and clinical response, such as referring patients for specialty care or social services.^13^ In 2012, the American Academy of Pediatrics (AAP) issued a recommendation that pediatricians should provide anticipatory guidance and actively screen for markers of toxic stress,^14^ similar to approaches to developmental screening^15^ and child behavioral health screening.^16^ Although surveys suggest most PCPs do not comprehensively inquire about ACEs,^11^, ^17^, ^18^ implementation research indicates that ACE screening with validated instruments is both feasible and acceptable.^19^, ^20^, ^21^, ^22^, ^23^, ^24^, ^25^, ^26^, ^27^, ^28^, ^29^, ^30^, ^31^, ^32^

ACE screening and clinical response in primary care could also come with challenges. For example, PCPs have reported insufficient time to incorporate new screenings and interventions.^33^^,^^34^ When asked about confidence screening for and responding to ACEs, PCPs convey hesitancy.^32^^,^^35^^,^^36^ Whether or not PCPs have been trained in approaches to trauma-informed care, engaging patients in conversation about ACEs could risk re-traumatization.^10^^,^^37^ This tension—between the perceived utility and challenges of addressing ACEs in primary care—raises a question as to whether ACE training, screening, and clinical response in primary care is associated positive and/or negative patient and provider outcomes. Answering this would inform clinicians and policymakers as to whether practices are commensurate with the existing science, or if more evidence is required.

To our knowledge, no studies have systematically reviewed the effects of implementing ACE training, screening, and clinical response in primary care settings for a composite of ACE exposures. In this study, we present findings from a systematic review of the peer-reviewed literature on this topic, in which studies identified forms of adversity as ACEs. Primary outcomes of interest were provider knowledge, confidence, screening behavior, and clinical care, and patient satisfaction, engagement in referrals, and health outcomes.

## Methods

### Search strategy and selection criteria

We searched the literature from January 1, 1998 through May 31, 2023 and reported results following PRISMA guidance.^38^ We interviewed content experts to inform our scope and identify search terms and Medical Subject Headings (MeSH) phrases and chose six inclusion/exclusion criteria that corresponded to the PICOTS framework.^39^ Specifically, studies needed to:

1. Pertain to one or more of the categories of ACEs described by the landmark CDC-Kaiser Permanente study^2^^,^^3^ and assess a composite of ACE exposures.
2. Focus on ACE training, screening, and/or response with respect to composite ACE measurement—using a tool such as the CDC-Kaiser Permanente Questionnaire—rather than measurement of a single ACE exposure such as child sexual abuse or intimate partner violence.
3. Involve implementation of ACE training, screening and/or response in a primary care setting, defined in terms of one of the following clinical areas: pediatrics, family medicine, internal medicine, obstetrics and gynecology, or geriatrics.
4. Focus on PCPs or staff typically present in primary care settings, rather than specialty providers.
5. Analyze the effects of implementation on one or more pre-specified provider and/or patient outcomes (see Data Abstraction and Synthesis).
6. Constitute empirical research that underwent peer review. We excluded commentaries, viewpoints, guidelines, conference abstracts and proceedings, and reviews.
7. Be written in or translated into English.

For a study to be included, it needed to contain one or more search terms within two domains: first, an indication the article pertained primary care and/or primary care providers; second, an indication the study focused provider and/or patient outcomes related to ACEs or toxic stressors that could arise during childhood or adolescence. These criteria were applied to four databases: PubMed, Web of Science, APA PsycInfo, and CINAHL. The complete search strategy can be found in Appendix I.

Each title and abstract were independently screened by two members using DistillerSR,^40^ allowing the team to calculate percent agreement among screeners. In the event of disagreement, a third team member served as a tiebreaker. For articles that inclusion was indeterminable, the full article was reviewed.

### Data analysis

Each article was assigned a primary and secondary abstracter: the primary abstracter was responsible for reviewing the article and abstracting data into a standardized form. The secondary abstracter reviewed accuracy and quality. We abstracted the following study characteristics: title, authors, publication year, geographic setting, study design, study period, study population(s), sample size(s), and provider-related outcomes and patient-related outcomes. We also clarified whether the study focused on pediatric or adult populations. Given that a large majority targeted pediatric populations, study findings are focused on children and adolescents unless explicitly stated otherwise in the Results or Discussion.

For the purposes of this review, assessments of ACEs included those focused on measurement of multiple ACEs, as captured by composite screening and assessment tools. We limited studies to those assessing composite ACE exposures because we were targeting articles that conscribed to the ACEs conceptual framework—i.e., that recognized that varying forms of childhood adversity converge on a common set of physiologic stress-response systems, and that this in turn may lead to negative health outcomes.

To be coded as an ACE training intervention, a study needed to describe implementation and measure outcomes associated with training PCPs and staff on the epidemiology of multiple ACEs, identification of multiple ACEs in clinical settings, and/or appropriate responses once ACEs were identified. To be coded as an ACE screening intervention, a study needed to describe implementation and measure outcomes associated with the introduction, scale-up, or strengthening of composite ACE screening in primary care settings. To be coded as an ACE response intervention, a study needed to describe implementation and measure outcomes associated with behaviors in response to identified ACEs in primary care. Studies could be coded to one, two, or all three intervention categories.

For provider-related outcomes, we indexed provider knowledge, confidence and self-efficacy, screening behavior, and clinical response—inclusive of changes in clinical care, reporting, and referral practices. For patient-related outcomes, we quantified acceptability and satisfaction, engagement with referrals, and health outcomes. To the extent possible, we reported measures indicating the magnitude of effects, strength of associations, and statistical significance.

We rated strength of evidence (SOE) according to GRADE criteria. Two team members independently assigned scores each article, based four domains: risk of bias, inconsistency, indirectness, and imprecision. Observational studies received a starting score of “low”, while randomized studies received a starting score of “high”. Studies could be upgraded or downgraded based on individual assessments within each domain. In the event two team members arrived at different scores, each evaluation was shown to all team members for collective deliberation. All team members collectively deliberated the weight of evidence across studies for each outcome of interest. SOE was assigned an ordinal value: very low (+), low (++), medium (+++), or high (++++).

### Role of the funding source

The funders of the study had no role in study design, data collection, data analysis, data interpretation, or writing of the report. RKM had access to all data and was responsible for the decision to submit for publication.

## Results

**Fig. 1:**
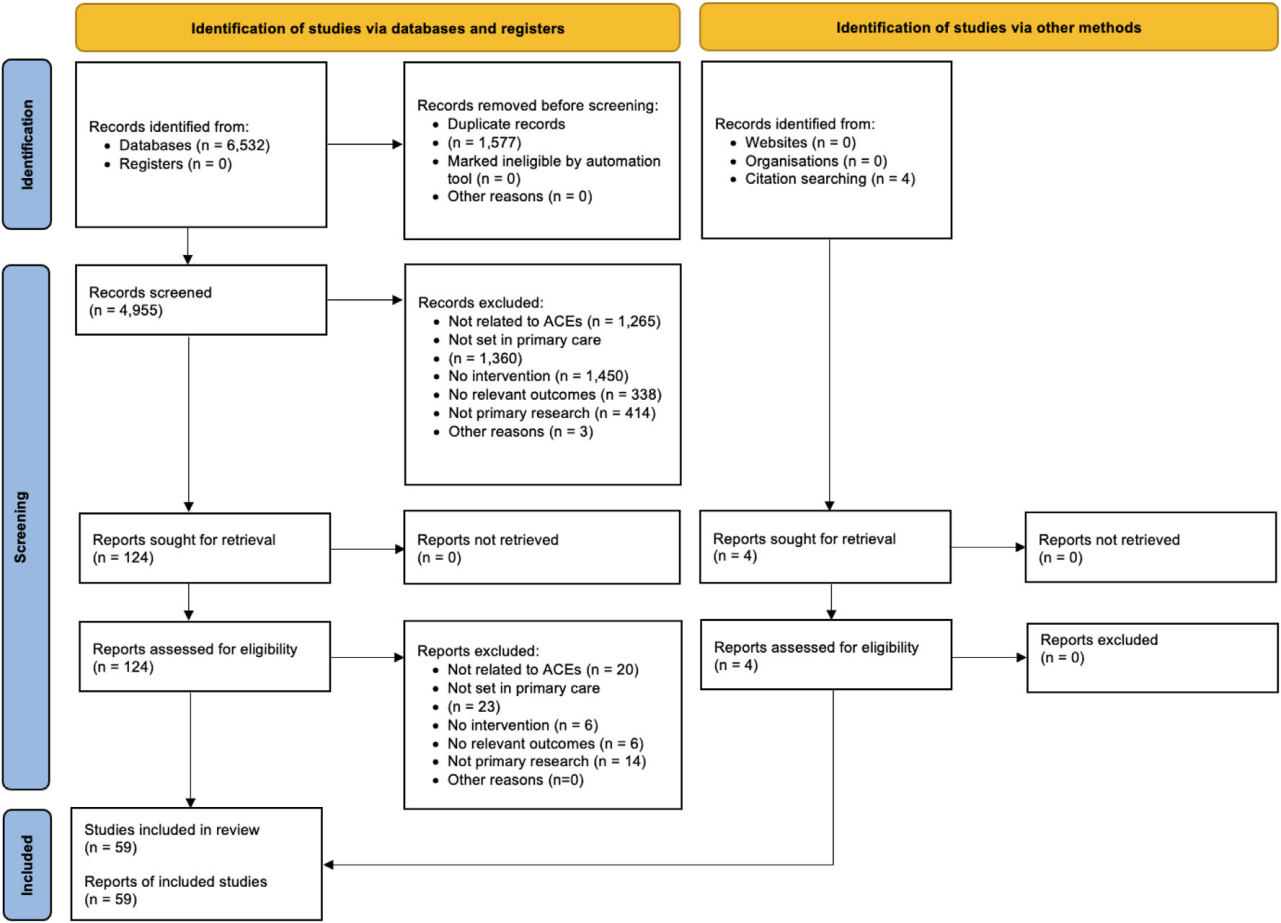
**Literature review flow diagram**. ACEs, adverse childhood experiences. Reasons for exclusion were identified in sequential order: i.e., reviewers first identified whether the study related to ACEs, and only if the study related to ACEs would they proceed to inspecting whether the study was set in primary care, etc.

The literature search generated 6532 articles, of which 1577 were duplicates, yielding 4955 unique studies (see Fig. 1). Of these, 4827 were excluded upon review of the title/abstract, leaving 128 articles for full-text retrieval. Of these, 58 met inclusion criteria. Percent agreement throughout the dual coding process was 96%, indicating strong overall concordance. A summary of studies is available in Appendix II.

Among included studies, 41 used samples from the United States, 3 from the Netherlands, two from the United Kingdom, and one each from Australia, Canada, Denmark, Japan, Kenya, and Saudi Arabia. Twelve studies did not list location. Studies contained an average of 73 providers and 1781 patients and represented a broad age range and both sexes. Seven studies were randomized controlled trials (RCTs) and two were quasi-experimental. Of the remaining, 20 used mixed methods, 13 used a prospective cohort design, 11 were cross-sectional, and seven were qualitative.

### Provider-related outcomes

#### ACE training

##### Knowledge

Ten of 13 studies in this category examined provider knowledge before versus after ACE training.^22^^,^^41^, ^42^, ^43^, ^44^, ^45^, ^46^, ^47^, ^48^, ^49^ Of these, eight found significant improvements in provider knowledge,^22^^,^^41^^,^^43^^,^^45^, ^46^, ^47^, ^48^, ^49^ including one RCT.^45^ Two recorded self-assessed knowledge gains^41^^,^^45^; the remaining employed external measurement sources, such as exams. The RCT^42^ and one pre-post study found no improvements.^44^ The remaining studies asked providers to self-report knowledge gains at a single time point after training,^50^, ^51^, ^52^, ^53^ with each study reporting significant improvements.

##### Confidence/self-efficacy

Eight of 15 studies in this category examined changes in confidence and/or self-efficacy from before to after training.^22^^,^^41^, ^42^, ^43^, ^44^, ^45^^,^^47^, ^48^, ^49^^,^^51^^,^^53^, ^54^, ^55^, ^56^, ^57^ All found large improvements. Four studies employed qualitative methods, with mixed feedback.^22^^,^^44^^,^^51^^,^^55^ In three, providers expressed a lack of confidence in screening and responding to ACEs. Two additional studies employed cross-sectional surveys: one found more training was associated with greater confidence^53^; the other measured confidence pre-training, with a majority (62%) expressing low confidence levels.

##### Screening behavior

All nine studies in this category—three of which were RCTs^42^^,^^45^^,^^58^—reported high rates of screening uptake after training.^22^^,^^42^^,^^43^^,^^45^^,^^48^^,^^51^^,^^57^, ^58^, ^59^ In five, screening rates exceeded 75%.^22^^,^^43^^,^^45^^,^^51^^,^^59^ In two others, rates remained below 50% but represented substantive improvements from baseline.

##### Clinical response

Five studies discussed changes in clinical care following training interventions.^41^^,^^42^^,^^46^^,^^51^^,^^57^ Two focused on an intervention known as Safe Environment for Every Kid (SEEK). The first found greater than 60% uptake in motivational interviewing after training.^51^ The other, an RCT, documented that providers in the intervention group more frequently addressed patients’ household difficulties, including parental stress, mental illness, and substance misuse.^42^ Two studies observed an increased frequency discussing ACEs with patients^41^ and providing interpersonal support.^46^ One qualitative study noted that providers felt screenings interrupt the clinical workflow.^57^

Four studies examined reporting and documentation practices, following training efforts. Three focused on child protective services (CPS). The first observed an increase in providers’ understanding of their reporting responsibilities following training^49^; the second did not find a change in frequency of reporting child abuse cases following training.^52^ The third conducted focus group discussions with providers, who reported that training helped foster a collaborative relationship with families to initiate CPS referrals.^60^ The remaining study focused on medical record documentation, finding a seven-fold increase after training in documenting patient-reported experiences of abuse.^61^

Lastly, four studies inspected changes in referral patterns after ACE training.^22^^,^^56^^,^^58^^,^^59^ Two examined pre-post changes in referrals, finding sizable improvements.^56^^,^^58^ One descriptive study found 47% of children who met criteria for referral were referred^59^ and another found 64% were provided a referral.^22^

#### ACE screening

##### Knowledge

Five studies found that screening pilots led to improvements in knowledge on effective ACE screening.^19^^,^^22^^,^^43^^,^^46^^,^^51^ Three studies characterized provider knowledge on ACE screening or child abuse,^26^^,^^62^^,^^63^ two of which found that most providers lacked knowledge or training on these topics.^26^^,^^62^

##### Confidence/self-efficacy

Of five studies that conducted pilot programs,^22^^,^^26^^,^^28^^,^^42^^,^^43^ four were associated with increased confidence levels.^22^^,^^26^^,^^42^^,^^43^ Three found that providers felt moderately confident screening for ACEs and that it helped them identify patient trauma.^62^^,^^64^^,^^65^ One study highlighted reasons for low provider confidence in ACE screening, such as lack of education, screening tools, or knowledge regarding local resources, and feelings of inadequacy managing suspected abuse.^51^ One qualitative study found that providers were more confident with a structured ACEs screener, though they were still concerned with false positives.^57^ Three found that providers felt moderately confident screening for ACEs and that it helped them identify patient trauma.^62^^,^^64^^,^^65^

##### Screening behavior

Of 24 articles measuring screening behavior, 20 evaluated interventions,^19^^,^^22^^,^^26^^,^^28^^,^^29^^,^^31^^,^^42^^,^^43^^,^^51^^,^^57^, ^58^, ^59^^,^^64^^,^^66^, ^67^, ^68^, ^69^, ^70^, ^71^ of which 19 identified screening rate improvements.^19^^,^^22^^,^^26^^,^^28^^,^^31^^,^^42^^,^^43^^,^^51^^,^^57^, ^58^, ^59^^,^^64^^,^^66^, ^67^, ^68^, ^69^, ^70^ At baseline, screening rates were close to zero in multiple instances; after the intervention, screening rates ranged from 23%^42^ to 100%.^19^ Two studies identified facilitators of screening uptake, such as lower screening burdens^72^ and use of face-to-face screenings.^67^ One study identified interrupted clinical workflows as a barrier to screening.^57^ Four studies examined existing ACE screening trends, three of which found a minority of patients were regularly screened,^62^^,^^63^^,^^72^ ranging from 17%^63^ to 47%.^72^

##### Clinical response

Of 12 studies that examined post-screening clinical care, which ranged from providing resources to motivational interviewing to treatment planning, six studies found either high rates of clinical responses or significant improvements after an intervention,^42^^,^^46^^,^^51^^,^^66^^,^^68^^,^^73^ whereas one study found no change in care delivery.^19^ Three studies conducted interviews with providers, one of which found that ACE disclosure directly informed treatment^64^; one found that screening was inconsistent before implementing a structured screener^57^; and the last found no consensus on clinical responses.^74^

Of 14 studies identifying referrals as a clinical response outcome, seven concluded implementation of screenings was associated with increased referrals.^29^^,^^58^^,^^64^^,^^68^^,^^69^^,^^75^^,^^76^ One RCT found that the intervention corresponded to 6.7 times higher adjusted odds of a referral,^58^ and another RCT found significantly lower odds of having a sick visit, ED visit, hospitalization.^56^ All others, which were observational, estimated effects from 13%^29^ to over 57%^77^ of patients referred after ACE screening.^19^^,^^29^^,^^58^^,^^64^^,^^68^^,^^75^, ^76^, ^77^

One study examined mandated reporting.^65^ This study assessed ACE conversations in 238 parent/guardian and 231 provider surveys and found that, while there were 20 disclosures of potential ACEs, none resulted in reporting.^65^

##### Rapport with patients

Five studies examined provider rapport with patients.^26^^,^^62^^,^^64^^,^^65^^,^^78^ One study surveyed primary care providers and found 71% of providers perceived guardians to be positive or receptive to discussing ACEs, 27% perceived guardians to be neutral, and 1% perceived guardians to be negative.^65^ Another study showed that providers were uncomfortable sharing inquiries about psychosocial issues and were concerned about offending patients.^62^ Three studies employed in-depth interviews. In two, providers expressed that screening improved their rapport with patients,^26^^,^^64^ and in one, parents recommended providers foster trust by explaining the process for screening and response.^78^

#### ACE response

##### Knowledge

One of seven articles^23^^,^^42^^,^^51^^,^^79^, ^80^, ^81^, ^82^ evaluated interventions for providers using trauma-informed care practices,^42^^,^^51^ of which one found improvements in knowledge of local social services to respond to ACEs.^51^ The other studies found that most providers possessed limited knowledge how to respond to ACEs,^79^, ^80^, ^81^ but knowledge increased with experience.^23^

##### Confidence/self-efficacy

Four of five studies^23^^,^^42^^,^^51^^,^^80^^,^^82^ were observational, two of which found that providers lacked confidence to screen for child abuse, respond to suspected abuse, and/or identify local resources to respond to ACEs.^51^^,^^80^ One qualitative article found that providers were uncomfortable reporting children to unknown entities.^82^ The RCT reported that participation in the SEEK model led providers to report greater comfort addressing ACEs.^42^

##### Clinical response

Of five studies that examined onsite clinical responses to ACEs, two evaluated Practicing Safety, finding that provider participation was associated with a higher likelihood of offering evidence-based guidance^68^ and posting community resources or patient education information. Two other studies, on the SEEK model, found that providers used motivational interviewing^51^ and demonstrated improvements in addressing depression, intimate partner violence, substance use, and stress.^42^ The remaining study surveyed providers on existing practices, finding that over 75% contacted on-site therapists to engage with their patients after screening for ACEs.^73^

We identified eight studies that examined external referrals as a clinical response.^23^^,^^56^^,^^58^^,^^68^, ^69^, ^70^^,^^75^^,^^76^ Five quantified referral rates, ranging from 28%^23^ to 57%^70^ of all patients in the study intervention group. Two additional studies, both evaluating Practicing Safety, found increases in referrals to social workers and community resources.^68^^,^^69^ An RCT found that the odds of receiving any referral and the odds of receiving a behavioral health agency referral both increased by a factor of 50%.^56^

Four of seven clinical response studies^68^^,^^80^^,^^83^^,^^84^ produced quantitative estimates on mandated reporting. One study found that 85% of pediatric professionals reported fewer than five cases per year.^80^ Another qualitative study found that providers, in deciding whether to file a report to CPS, felt tension between meeting the needs of caregivers and young persons experiencing abuse.^83^

##### Rapport with patients

Three studies examined provider-patient rapport.^78^^,^^82^^,^^83^ Two study found concerns that reporting suspected abuse changed the provider's relationship with the patient's family.^82^^,^^83^ The third, a qualitative study, had parents offer guidance to providers: including to foster trust by explaining the ACE screening process.^78^

#### Strength of evidence

SOE assessments of individual studies can be found in Appendix Table 3. With three exceptions, SOE for provider-related outcomes was very low or low (Table 1). Contributing factors were a paucity of RCTs, conflicting findings, reliance on self-reported measures, and frequent use of surveys subject to non-response and social desirability bias. One exception was the effect of ACE training on confidence/self-efficacy, for which 15 studies were included. All eight pre-post studies found improvements in confidence following training, with medium-to-large effect sizes. A second exception pertained to interventions aimed at expanding ACE screening. Most studies in this category identified significant improvements in screening rates, although sample sizes were often small. The third exception related to ACE response interventions’ effect on clinical response, particularly connecting patients to services. These studies found significant increases in referral rates.

**Table 1:** Strength of evidence: provider-related outcomes.

Outcome	ACE Training	ACE Screening	ACE Response
Knowledge	Low (n = 13): Two RCTs, one of which reported improvements and one did not find a group difference; observational studies found modest pre-post improvements and four found no effect or mixed results; self-reported knowledge gains subject to social desirability bias; most studies reported small sample sizes and were subject to non-response bias on surveys.	Low (n = 9): All studies contained small sample sizes and no comparison groups; most did not use statistical methods to control for confounding variables.	Very Low (n = 7): Only one study quantified an improvement knowledge; all studies were observational, had small sample sizes, and lacked comparison groups.
Confidence/Self-Efficacy	Moderate (n = 15): All eight pre-post assessments found improvements; magnitude of effects was often sizeable; sample sizes were generally small; surveys had potential for non-response bias and social desirability bias; one RCT found improvements in confidence that persisted for 18 months.	Low (n = 10): All studies were observational and without comparison groups; provider responses were self-reported and subject to social desirability bias; largely inconsistent findings as to whether interventions led to improved confidence.	Very Low (n = 8): Nearly all studies, including an RCT, either found no improvements or generally low confidence levels when responding to ACEs; most studies were observational.
Screening Behavior	Moderate (n = 9): The eight studies involving inferential analyses all found significant improvements, including three RCTs; the magnitude of reported effects was generally large; most studies were observational with small sample sizes; surveys had potential for non-response bias and social desirability bias.	Moderate (n = 24): Most studies found large estimates of improvements in screening rates; most sample sizes were small, relied on observational analyses, and did not have comparison groups; survey responses were subject to recall and social desirability bias.	N/A (n = 0)
Clinical Response	Low (n = 13): Only 5 studies examined clinical care, 5 examined referrals, and 4 examined reporting practices; evidence of measuring outcomes indirectly (e.g., reporting responsibilities rather than reporting behavior); studies were predominately observational.	Moderate (n = 26): Studies were largely observational, except for two RCTs; most did not have control groups and contained small sample sizes; most studies identified small clinical responses to ACEs.	Moderate (n = 17): Studies documented large changes; most sample sizes were small, lacked comparison groups and used observational methods.
Rapport	N/A (n = 0)	Very Low (n = 5): Small number of studies; all observational; subject to recall and social desirability bias.	Very Low (n = 3): Studies used observational methods and small sample sizes; rapport between providers and patients was estimated to be low.

ACE, Adverse childhood experience. RCT, Randomized controlled trial. Options for strength of evidence were: very low, low, medium, and high, based on GRADE criteria.

### Patient-related outcomes

#### ACE training

##### Acceptability/satisfaction

Three of five studies in this category were RCTs. One found that patients served by providers randomized to training reported significantly greater satisfaction with their visit.^45^ The second found that patients who interacted with providers randomized to receive training reported a 67% reduction in unmet desires to discuss psychosocial issues.^58^ Three studies, one RCT and two observational, found that patients or caregivers were more comfortable discussing ACEs with their provider, subsequent to provider training.^51^^,^^57^^,^^61^

##### Engagement in referrals

Of two studies in this category, one RCT found caregivers served by providers assigned to training were 17-times more likely to contact community resources.^58^ The second, an observational study, found referral acceptance rates ranged from 14% to 30% following provider training, depending on the facility.^51^

##### Health outcomes

Two RCTs found that caregivers who were served by providers assigned to the training group reported fewer instances of psychological aggression or physical violence, compared to those served by providers in the control group.^85^^,^^86^

#### ACE screening

##### Acceptability/satisfaction

Eleven of 14 studies in this category comprised surveys (n = 10) or interviews (n = 1) of caregivers or patients after screening.^22^^,^^27^^,^^29^^,^^57^^,^^58^^,^^61^^,^^65^^,^^77^^,^^78^^,^^87^^,^^88^ In all instances, individuals reported high levels of satisfaction/acceptability—ranging from 65%^88^ to 94%.^77^ However, one study found that while overall acceptance was high, over half of the Native American and Hispanic/Latinx did not consider screening to be acceptable.^88^ Two studies asked providers to assess whether ACE screening appeared to be acceptable to patients. In both instances, providers affirmed acceptability.^19^^,^^51^

##### Engagement in referrals

Among five studies examining referral acceptance,^26^^,^^51^^,^^58^^,^^77^^,^^89^ three reported acceptance rates, ranging from a low of 14% at an FQHC^51^ to a high of 77% at a pediatric clinic serving predominately low-income families.^26^ Two studies, one RCT^58^ and one quasi-experimental study,^89^ found caregivers or children served by providers assigned to the screening intervention were 17-times and 7.5-times, respectively, more likely to contact community or behavioral health resources.

##### Health outcomes

Of three studies, two RCTs focused on reductions in household violence. Each found that physical violence or psychological aggression was reduced among parents served by intervention providers.^85^^,^^86^ One prospective cohort study measured outcomes before versus after screening for and responding to ACEs—using a motivation-based intervention.^77^ The authors found short-term effects on self-reported unhealthy alcohol use and risky sexual behavior, as well as sustained effects on perceived stress.

#### ACE response

##### Acceptability/satisfaction

Five observational studies examined patient satisfaction. One reported high levels of satisfaction (>90%) with an intervention involving motivational interviewing^77^; a second found a plurality of caregivers felt intervention content was helpful^90^; a third interviewed providers, who shared that caregivers generally appeared comfortable discussing psychosocial risk factors for maltreatment^51^; a fourth found that families and patients were open to discussing ACEs and toxic stress^87^; the fifth reported that parents understood the importance of discussing and responding to ACEs.^78^ One RCT found parents in the intervention group had fewer unmet needs to discuss psychosocial issues than those in the control group.^58^

##### Engagement in referrals

Two observational studies examined acceptance of referrals. One found 39% of patients accepted behavioral health referrals one-month post-intervention.^77^ The second found that 14%–30% of patients accepted referrals to specialty care, depending on the clinical setting.^51^ One RCT found parents in the intervention group had significantly higher likelihood of contacting community services.^58^ Similarly, one quasi-experimental study found children in the intervention group were more likely to schedule and visit a behavioral health care provider.^89^

##### Health outcomes

Three studies reported patient health outcomes.^77^^,^^85^^,^^86^ One study found that motivational interviewing in response to identified ACEs was associated with short-term reductions in self-reported unhealthy alcohol use and nutrition habits and risky sexual behavior.^77^ Lastly, two RCTs documented reductions in household violence following the intervention, as self-reported by caregivers.^85^^,^^86^

#### Strength of evidence

SOE assessments of individual studies can be found in Appendix Table 3. For all outcomes save one, SOE pertaining to patient-related outcomes was either very low or low (Table 2). Chief reasons were: most studies were observational, results were based on small sample sizes, and authors relied on self-reported survey data. One exception was the relationship between interventions that targeted improvements in ACE screening rates and patient-reported acceptability and satisfaction, for which there was moderate strength of evidence. All studies in this category reported high levels of acceptability and satisfaction—typically above 90%.

**Table 2:** Strength of evidence associating interventions with patient-related outcomes.

Outcome	ACE Training	ACE Screening	ACE Response
Acceptability/Satisfaction	Low (n = 5): All studies found high levels of acceptability and satisfaction, including two RCTs; only five studies, inspecting different—albeit related—outcomes; studies had potential for social desirability bias.	Moderate (n = 14): Studies were predominately observational; outcomes were self-reported; respondents (patient, caregiver, provider) varied across studies; most studies recorded high levels of satisfaction and acceptability.	Very Low (n = 5): Four of five studies were observational; outcomes were self-reported; sample sizes were small; all findings indicated positive feedback from patients and caregivers.
Engagement in Referrals	Very Low (n = 2): Small number of studies on the topic, with only one RCT; acceptance of referrals was as low as 14% in one setting; both studies provided quantitative estimates for referral acceptance.	Very Low (n = 5): Small number of studies; sample sizes were modest; observed acceptance of referral rates ranged from 14% to 77%; one RCT and one quasi-experimental study both found a large increase in patients accessing community resources among those in intervention group.	Very Low (n = 4): Small number of studies; two studies were observational; small sample sizes; low acceptance rates (<40%) of referrals in both studies.
Health Outcomes	Very Low (n = 2): Only two studies; outcomes were self-reported and may be subject to social desirability bias.	Low (n = 3): Studies examined limited set of outcomes; two RCTs found an intervention (SEEK) reduced household violence, though outcome was self-reported; observational study found effects that largely dissipated within two months.	Very Low (n = 3): Small number of studies; outcomes were self-reported; improvements largely dissipated within two months.

ACE, Adverse childhood experience. RCT, Randomized controlled trial. Options for strength of evidence were: very low, low, medium, and high, based on GRADE criteria.

## Discussion

This systematic review indicates that evidence on the benefits of expanding ACE training, screening, and response in primary care remains nascent. Where available, studies have typically indicated positive associations. Yet, for the most part, we failed to observe consistent linkages between specific interventions and outcomes. Additionally, studies were predominately observational and subject to a variety of limitations.

With respect to ACE training interventions, a preponderance of studies focused on self-reported changes in provider knowledge and confidence. In most instances, studies recorded positive, short-term gains. Whether gains were maintained, and the extent to which they altered clinical practice, were unclear. Thirteen studies—including two RCTs—also reported large increases in ACE screening rates following training. The magnitude of these gains was comparable to documented rates on the scale-up of behavioral health screenings more generally.^91^^,^^92^ Fifteen studies quantified changes in how providers responded to identified ACEs following training interventions. In all instances save one,^52^ training was associated with positive changes. For example, an RCT conducted by Garg and colleagues (2007) found a fivefold increase in referrals among providers who received the training intervention.^58^

A common pattern here and elsewhere pertained to lack of insight on referral practices. In a recent analysis, Hartveit and colleagues (2017) articulate several quality indicators for assessing referral processes, including whether the referral is appropriate and results in timely access to care.^93^ Among studies in this review quantifying referrals, these quality indicators were largely omitted—leaving an open question as to whether referrals benefited patients in a meaningful way. Future studies on referrals from ACE screening in primary care might pursue this line of inquiry.

Only nine studies examined the association between ACE training interventions and patient-related outcomes, primarily patient satisfaction with care and acceptability of ACE screening. Three RCTs found patients in intervention arms reported higher levels of comfort,^61^ satisfaction,^45^ and fewer unmet needs. Among the two studies measuring patient engagement in referrals, results varied considerably: from a 17-fold increase in acceptance of referrals to only marginal adoption.^51^^,^^58^ This lack of consistency in effect sizes was a common trend across studies, reducing strength of evidence (SOE). Two studies, both RCTs, found that parents who interacted with trained providers reported fewer instances of psychological aggression or physical violence directed towards their children.^85^^,^^86^ This is consistent with past research on the training benefits for clinical social workers and therapists in specialty care settings.^94^^,^^95^ However, these studies relied on self-reporting that are subject to social desirability bias. Additional research would benefit from parent-child dyadic measurement, which cross-validates parent and child responses.^96^

With respect to the 29 screening interventions we reviewed, 25 quantified subsequent changes in providers’ screening behavior. Collectively, these studies provided medium SOE, similar to the quality of evidence and risk of bias documented in previous systematic reviews on uptake of screening for cancers and mental health conditions.^97^, ^98^, ^99^ All but two were observational; however, the magnitude of uptake in screening rates following interventions was consistently large. Remaining outcomes associated with screening interventions possessed low SOE, the product of small sample sizes, lack of comparison groups, and inconsistent findings.

Twenty-one studies examined how ACE screening interventions influenced patient-related outcomes. Fifteen quantified patient or caregiver acceptability and/or satisfaction, reporting high levels in each category—ranging from 72%^61^ to 94%.^77^ Nonetheless, SOE was low: studies were observational and subject to biases such as non-response bias and social desirability bias. Of five studies examining patient engagement in referrals, three reported a wide range of referral acceptance rates, from 14%^51^ to 77%.^26^ Only three studies examined patient health outcomes, two of which were RCTs. Both RCTs found that implementation of a specific intervention (SEEK) corresponded with reduced household violence.^85^^,^^86^ Across all interventions we identified, SEEK possessed the broadest corpus of evidence.^42^^,^^45^^,^^51^^,^^85^^,^^86^

With respect to ACE response interventions, we only identified seven that addressed provider-related outcomes. Three outcomes—provider knowledge, confidence, and rapport with patients—possessed very low SOE: multiple studies found little or no relationship with improved knowledge,^42^^,^^79^, ^80^, ^81^ and a similar number reported low levels of confidence in providers’ abilities to execute screening and response.^51^^,^^100^^,^^101^ Similarly, one qualitative study documented that providers were hesitant to discuss ACEs or take action following identification of issues like suspected physical abuse because of the potential to compromise the therapeutic relationship with families.^83^ This is the closest we observed to distress or discomfort resulting from ACE screening and response in primary care; however, neither distress nor discomfort were objectively measured, which could be a valuable pursuit in future research.

A larger group of studies (n = 14) with moderate SOE found ACE response interventions led to meaningful changes in clinical care and increased referrals. For example, Gerlach and colleagues (2021) found that—following the ACE intervention—over three-quarters of providers reached out to on-site clinical social workers or therapists to engage with patients after positive screening.^73^

It was uncommon for studies to document ways in which altered provider behavior affected patient outcomes: we identified only seven studies, all focused on patient satisfaction or acceptability. In most instances, feedback was positive.^51^^,^^58^^,^^75^^,^^77^^,^^78^^,^^87^^,^^90^ Two also looked at patients’ engagement of in referrals. Referral acceptance was below 50% in both cases.^51^^,^^77^ Prior studies in primary care settings have found that acceptance of referrals is influenced by wait times, proximity to care, provider scheduling of the referral, and the strength of the patient-provider relationship.^102^, ^103^, ^104^ It is also possible some referrals may have been unnecessary, leading patients to decline services.

Only 12 studies specifically focused on ACE screening and response among adults, compared to 50 focused on pediatric services (see Appendix 2, Tables C and D). Adult-focused studies were also lower strength of evidence: two studies of adults were medium or high SOE, compared to 9 studies of children that were medium or high. Studies among adults covered fewer domains of ACEs screening, training, and response. In addition, the study design for studies with children were stronger, including more RCTs. That said, observed patterns were similar: for example, studies found that provider knowledge and confidence increased after training,^22^^,^^23^ referrals increased as part of clinical response,^56^^,^^58^^,^^68^^,^^89^ and patients reported moderate to high levels of acceptability.^19^^,^^22^^,^^88^

Overall, our analysis indicates a critical gap in knowledge regarding the association between ACE training, screening, and response in primary care and provider- and patient-related outcomes. Studies were inconsistent in terms of the ACEs they investigated, interventions they employed, and outcomes they measured. Studies were also inconsistent in their own terminology, and we excluded studies that did not include ACE-specific language. Future studies that aim to be more comprehensive—by including studies that do not self-ascribe as focused on ACEs—would benefit from collating an inventory of clinical screeners that assess ACEs. This could then be used as the basis for future assessments of the ACEs literature.

Of the 58 studies we identified, only seven were RCTs. Among the observational studies, roughly half employed survey instruments that involved self-reporting, often with small sample sizes and low response rates. A strategic next step would be to generate expert consensus on which interventions in primary care may be the most likely to mitigate the sequelae of specific adversities, and then conduct experimental investigations. This includes investigations that employ implementation science frameworks to map relationships between processes and outcomes in the context of clinical practice. It also includes investigations in resource-limited settings where adversities—particularly economic and food insecurity—may be widely prevalent but are significantly understudied.

We note several study limitations. First, although our search strategy assessed over 6000 articles, it is possible we failed to capture relevant studies. We reviewed studies that self-identified as focused on training, screening, and response to multiple ACEs, rather than the broader literature on toxic stress physiology and pediatric clinical screeners, or the narrower literature on individual exposures such as child sexual abuse. Second, heterogeneity of sampling frames and study designs limited our ability to draw broad inferences. Likewise, interventions may have entailed components that were not explicitly described in the text and we were therefore unable to reflect particular nuances. Lastly, to the extent publication bias influenced this investigation, it would imply that strength of evidence may be weaker than that conveyed by our assessment.

Notwithstanding these limitations, this systematic review is the first we know of to show that high-quality information on the effects of ACE-related interventions in primary care settings is limited, and that the field is currently relying primarily on observational studies that have small sample sizes. At the same time, the extant literature indicates high rates of patient acceptability, improvements in provider knowledge and referrals, and reductions in household violence following specific interventions. We did not find any evidence indicating that ACE screening and response led to adverse outcomes, apart from possible tensions in caregiver-provider rapport when discussing ACEs—particularly in the context of suspected child abuse. Moving forward, researchers should endeavor to conduct rigorous studies to evaluate best practices for addressing the health consequences of ACE exposure.

## Contributors

RKM, NKE, JSL, and GMS conceived and designed the study. SM and NQ led data extraction, with FKM and JSL verifying extracted studies. Differences in opinion on study inclusion were resolved by discussion among RKM, NKR, JSL, SM, and NQ. RKM, SM, NQ, and JSL accessed and verified the underlying data. All authors (RKM, JSL, SM, NQ, DL, AS, RG, KK, GMS, and NKE) contributed to interpretation of the results and manuscript writing, and they approved the final manuscript for submission.

## Data sharing statement

All extracted data that correspond to the findings of this systematic review will be made available upon request after approval of a proposal from the corresponding author, beginning the publication date of this article (rmcbain@rand.org).

## Declaration of interests

RKM, NQ, DL, GMS, and NKE declare funding support from the California Department of Health Care Services over the prior 36 months, including since the initial planning of work. AS declares funding support from the Eunice Kennedy Shriver National Institute of Child Health & Human Development over the prior 36 months. RG declares funding support from UCLA-UCSF ACEs Aware and Family Resilience Network (UCAAN) over the prior 36 months, including since the initial planning of work, as well as consulting fees from UCAAN. RG also declares consulting fees from the Center for Youth Welnness and payment for expert testimony from Eglet Adams and Nevada State ACEs and Toxic Stress Abatement Plan. RG also declares participation in three data safety monitoring/advisee boards: for Pathways to Resilience Expert Advisory Committee–Aurrera Health, Expert Review Panel, Safe Spaces: Foundations of Trauma-Informed Practice for Educational and Care Settings–Office of the California Surgeon General, and National Early Relational Health Network. RG declares roles as the co-founder of the National Committee on Asthma and Toxic Stress and board membership of the Board Member, California American Professional Society on the Abuse of Children, as well as stock options in Stronger Brains Inc. KK declares support from California Department of Health Care Services and UCAAN since the initial planning of the work, as well as grants/contracts and consulting fees through Aurrera Health Group, Hillcrest Advisory, and the Population Health Innovation Lab in the prior 36 months.

## References

[1] Centers for Disease Control and Prevention (2022). https://www.cdc.gov/violenceprevention/aces/fastfact.html.

[2] Felitti V.J., Anda R.F., Nordenberg D. (1998). Relationship of childhood abuse and household dysfunction to many of the leading causes of death in adults. The Adverse Childhood Experiences (ACE) Study. Am J Prev Med.

[3] (2021). About the CDC-Kaiser ACE study.

[4] Franke H.A. (2014). Toxic stress: effects, prevention and treatment. Children.

[5] Hughes K., Bellis M.A., Hardcastle K.A. (2017). The effect of multiple adverse childhood experiences on health: a systematic review and meta-analysis. Lancet Public Health.

[6] Brown D.W., Anda R.F., Tiemeier H. (2009). Adverse childhood experiences and the risk of premature mortality. Am J Prev Med.

[7] Lorenc T., Lester S., Sutcliffe K., Stansfield C., Thomas J. (2020). Interventions to support people exposed to adverse childhood experiences: systematic review of systematic reviews. BMC Public Health.

[8] Ford D.E. (2017). The community and public well-being model: a new framework and graduate curriculum for addressing adverse childhood experiences. Acad Pediatr.

[9] Gill M.E., Zhan L., Rosenberg J., Breckenridge L.A. (2019). Integration of adverse childhood experiences across nursing curriculum. J Prof Nurs.

[10] Grossman S., Cooper Z., Buxton H. (2021). Trauma-informed care: recognizing and resisting re-traumatization in health care. Trauma Surg Acute Care Open.

[11] Keeshin B., Byrne K., Thorn B., Shepard L. (2020). Screening for trauma in pediatric primary care. Curr Psychiatry Rep.

[12] Beck R.S., Daughtridge R., Sloane P.D. (2002). Physician-patient communication in the primary care office: a systematic review. J Am Board Fam Pract.

[13] Dubowitz H., Finkelhor D., Zolotor A., Kleven J., Davis N. (2022). Addressing adverse childhood experiences in primary care: challenges and considerations. Pediatrics.

[14] Garner A.S., Shonkoff J.P. (2012). Committee on psychosocial aspects of child and family health, committee on early childhood, adoption, and dependent care, section on developmental and behavioral pediatrics. Early childhood adversity, toxic stress, and the role of the pediatrician: translating developmental science into lifelong health. Pediatrics.

[15] Balasundaram P., Avulakunta I.D. (2023). StatPearls.

[16] Jellinek M.S., Murphy J.M., Robinson J., Feins A., Lamb S., Fenton T. (1988). Pediatric Symptom Checklist: screening school-age children for psychosocial dysfunction. J Pediatr.

[17] Szilagyi M., Kerker B.D., Storfer-Isser A. (2016). Factors associated with whether pediatricians inquire about parents' adverse childhood experiences. Acad Pediatr.

[18] Kerker B.D., Storfer-Isser A., Szilagyi M. (2016). Do pediatricians ask about adverse childhood experiences in pediatric primary care?. Acad Pediatr.

[19] Glowa P.T., Olson A.L., Johnson D.J. (2016). Screening for adverse childhood experiences in a family medicine setting: a feasibility study. J Am Board Fam Med.

[20] Purewal S.K., Bucci M., Wang L.G. (2016). Screening for adverse childhood experiences (ACEs) in an integrated pediatric care model. Zero Three.

[21] Conn A.M., Szilagyi M.A., Jee S.H., Manly J.T., Briggs R., Szilagyi P.G. (2018). Parental perspectives of screening for adverse childhood experiences in pediatric primary care. Fam Syst Health.

[22] Flanagan T., Alabaster A., McCaw B., Stoller N., Watson C., Young-Wolff K.C. (2018). Feasibility and acceptability of screening for adverse childhood experiences in prenatal care. J Womens Health (Larchmt).

[23] Kalmakis K.A., Shafer M.B., Chandler G.E., Aponte E.V., Roberts S.J. (2018). Screening for childhood adversity among adult primary care patients. J Am Assoc Nurse Pract.

[24] Koita K., Long D., Hessler D. (2018). Development and implementation of a pediatric adverse childhood experiences (ACEs) and other determinants of health questionnaire in the pediatric medical home: a pilot study. PLoS One.

[25] Choi K.R., McCreary M., Ford J.D., Rahmanian Koushkaki S., Kenan K.N., Zima B.T. (2019). Validation of the traumatic events screening inventory for ACEs. Pediatrics.

[26] Kia-Keating M., Barnett M.L., Liu S.R., Sims G.M., Ruth A.B. (2019). Trauma-responsive care in a pediatric setting: feasibility and acceptability of adverse childhood experiences (ACEs) screening. Am J Community Psychol.

[27] Marie-Mitchell A., Lee J., Siplon C., Chan F., Riesen S., Vercio C. (2019). Implementation of the whole child assessment to screen for adverse childhood experiences. Glob Pediatr Health.

[28] Marsicek S.M., Morrison J.M., Manikonda N., O'Halleran M., Spoehr-Labutta Z., Brinn M. (2019). Implementing standardized screening for adverse childhood experiences in a pediatric resident continuity clinic. Pediatr Qual Saf.

[29] Selvaraj K., Ruiz M.J., Aschkenasy J. (2019). Screening for toxic stress risk factors at well-child visits: the addressing social key questions for health study. J Pediatr.

[30] Young-Wolff K.C., Alabaster A., McCaw B. (2019). Adverse childhood experiences and mental and behavioral health conditions during pregnancy: the role of resilience. J Womens Health (Larchmt).

[31] DiGangi M.J., Negriff S. (2020). The implementation of screening for adverse childhood experiences in pediatric primary care. J Pediatr.

[32] Gillespie R.J. (2019). Screening for adverse childhood experiences in pediatric primary care: pitfalls and possibilities. Pediatr Ann.

[33] Lipkin P.H., Macias M.M., Baer Chen B. (2020). Trends in pediatricians' developmental screening: 2002-2016. Pediatrics.

[34] Sprague S., Madden K., Simunovic N. (2012). Barriers to screening for intimate partner violence. Women Health.

[35] Lee A., Coles J., Lee S., Kulkarni J. (2012). Primary healthcare practitioners' screening practices and attitudes towards women survivors of child abuse. Ment Health Fam Med.

[36] Caneira L., Myrick K.M. (2015). Diagnosing child abuse: the role of the nurse practitioner. J Nurse Pract.

[37] (2015). Recognizing and treating child traumatic stress.

[38] Page M.J., McKenzie J.E., Bossuyt P.M. (2021). The PRISMA 2020 statement: an updated guideline for reporting systematic reviews. BMJ.

[39] Riva J.J., Malik K.M.P., Burnie S.J., Endicott A.R., Busse J.W. (2012). What is your research question? An introduction to the PICOT format for clinicians. J Can Chiropr Assoc.

[40] DistillerSR (2021). https://www.evidencepartners.com.

[41] Schmitz A., Light S., Barry C., Hodges K. (2019). Adverse childhood experiences and trauma-informed care: an online module for pediatricians. MedEdPORTAL.

[42] Dubowitz H., Lane W.G., Semiatin J.N., Magder L.S., Venepally M., Jans M. (2011). The safe environment for every kid model: impact on pediatric primary care professionals. Pediatrics.

[43] Bryant C., VanGraafeiland B. (2020). Screening for adverse childhood experiences in primary care: a quality improvement project. J Pediatr Health Care.

[44] Wilson C.R., Sherritt L., Knight J.R. (2005). Teaching residents about child neglect and parental alcoholism: a controlled pilot study. Med Educ Online.

[45] Feigelman S., Dubowitz H., Lane W., Grube L., Kim J. (2011). Training pediatric residents in a primary care clinic to help address psychosocial problems and prevent child maltreatment. Acad Pediatr.

[46] Froula L.M., Lenane A.M., Pasternack J.R., Garfunkel L.C., Baldwin C.D. (2017). Case-based workshop for teaching child abuse prevention to resident physicians. MedEdPORTAL.

[47] Berg-Poppe P., Anis Abdellatif M., Cerny S., LaPlante K., Merrigan M., Wesner C. (2022). Changes in knowledge, beliefs, self-efficacy, and affective commitment to change following trauma-informed care education for pediatric service providers. Psychol Trauma.

[48] Chokshi B., Chen K.L.D., Beers L. (2020). Interactive case-based childhood adversity and trauma-informed care electronic modules for pediatric primary care. MedEdPORTAL.

[49] Bannon M., Carter Y., Jackson N., Pace M., Thorne W. (2001). Meeting the training needs of GP registrars in child abuse and neglect. Child Abuse Rev.

[50] Henry B.M., Ueda R., Shinjo M., Yoshikawa C. (2003). Health education for nurses in Japan to combat child abuse. Nurs Health Sci.

[51] Eismann E.A., Theuerling J., Maguire S., Hente E.A., Shapiro R.A. (2019). Integration of the safe environment for every kid (SEEK) model across primary care settings. Clin Pediatr.

[52] Khan A.N.G.A., Rubin D.H., Winnik G. (2005). Evaluation of the mandatory child abuse course for physicians: do we need to repeat it?. Publ Health.

[53] Dara J.S., Kotlar E.Y., Leekoff M.L., Tran X.G., McColgan M.D., Giardino A.P. (2013). Resident comfort level after receiving child abuse training: a survey of pediatric chief residents. Child Abuse Negl.

[54] Lloyd M., Ratner J., La Charite J. (2021). Addressing trauma and building resilience in children and families: standardized patient cases for pediatric residents. MedEdPORTAL.

[55] Jee S.H., Conn A.M., Milne-Wenderlich A. (2020). Providing trauma-informed pediatric care for underserved populations: reflections on a teaching intervention. Dev Child Welfare.

[56] Eismann E.A., Zhang B., Fenchel M. (2023). Impact of screening and Co-located parent coaching within pediatric primary care on child health care use: a stepped wedge design. Prev Sci.

[57] Rosado J.I., Reyes E., Montgomery J., Wang Y., Malloy C., Simpson-O’Reggio A.M. (2023). From planning to implementation: developing an ACE screening protocol in a rural integrated primary care clinic serving Latino children. Clin Pract Pediatr Psychol.

[58] Garg A., Butz A.M., Dworkin P.H., Lewis R.A., Thompson R.E., Serwint J.R. (2007). Improving the management of family psychosocial problems at low-income children's well-child care visits: the WE CARE Project. Pediatrics.

[59] DiGiovanni S.S., Hoffmann Frances R.J., Brown R.S. (2023). Pediatric trauma and posttraumatic symptom screening at well-child visits. Pediatr Qual Saf.

[60] Campbell K.A., Wuthrich A., Norlin C. (2020). We have all been working in our own little silos forever: exploring a cross-sector response to child maltreatment. Acad Pediatr.

[61] Carroll J.C., Reid A.J., Biringer A. (2005). Effectiveness of the Antenatal Psychosocial Health Assessment (ALPHA) form in detecting psychosocial concerns: a randomized controlled trial. CMAJ.

[62] Alhowaymel F.M., Alzahrani N.S., Alharbi H.F., Almarwani A.M. (2023). Healthcare providers screening for childhood abuse among adult patients in Saudi Arabia: a cross-sectional study. J Nurs Scholarsh.

[63] Walbeehm-Hol L.K.M., Busari J.O. (2022). Awareness of toxic stress and adverse childhood experiences among Dutch pediatric health care providers: a national survey. Clin Med Res.

[64] Liu S.R., Grimes K.E., Creedon T.B., Pathak P.R., DiBona L.A., Hagan G.N. (2021). Pediatric ACES assessment within a collaborative practice model: implications for health equity. Am J Orthopsychiatry.

[65] Bodendorfer V., Koball A.M., Rasmussen C., Klevan J., Ramirez L., Olson-Dorff D. (2020). Implementation of the adverse childhood experiences conversation in primary care. Fam Pract.

[66] Chung E.K., Gubernick R.S., LaNoue M., Abatemarco D.J. (2019). Child abuse and neglect risk assessment: quality improvement in a primary care setting. Acad Pediatr.

[67] Crenshaw M.M., Owens C.R., Dow-Smith C., Olm-Shipman C., Monroe R.T. (2021). Lessons learned from a quality improvement initiative: adverse childhood experiences screening in a pediatric clinic. Pediatr Qual Saf.

[68] Abatemarco D.J., Gubernick R.S., LaNoue M.D. (2018). Practicing safety: a quality improvement intervention to test tools to enhance pediatric psychosocial care for children 0-3 years. Prim Health Care Res Dev.

[69] Abatemarco D.J., Kairys S.W., Gubernick R.S., Kairys J.A. (2008). Expanding the pediatrician's black bag: a psychosocial care improvement model to address the “new morbidities”. Jt Comm J Qual Patient Saf.

[70] Brennan L., Evans M., Michaeli G. (2022). Completion of social drivers of health screenings in pediatric practices participating in a quality improvement initiative. J Dev Behav Pediatr.

[71] Konijnendijk A.A.J., Boere-Boonekamp M.M., Haasnoot M.E., Need A. (2019). Effects of a computerised guideline support tool on child healthcare professionals' response to suspicions of child abuse and neglect: a community-based intervention trial. BMC Med Inform Decis Mak.

[72] Popp T.K., Geisthardt C., Bumpus E.A. (2020). Pediatric practitioners' screening for adverse childhood experiences: current practices and future directions. Soc Work Public Health.

[73] Gerlach B., LaBrenz C.A., Barczyk A.N. (2021). ACE-informed responses in central Texas: findings from a needs assessment. Soc Work Public Health.

[74] Reading J., Nunez D., Torices T., Schickedanz A. (2022). A qualitative study of pediatricians' adverse childhood experiences screening workflows. Acad Pediatr.

[75] Quizhpi C., Schetzina K., Wood D. (2019). Breaking the cycle of childhood adversity through pediatric primary care screening and interventions: a pilot study. Int J Child Health Hum Dev.

[76] Yaun J.A., Rogers L.W., Marshall A., McCullers J.A., Madubuonwu S. (2022). Whole child well-child visits: implementing ACEs and SDOH screenings in primary care. Clin Pediatr.

[77] Goldstein E., Topitzes J., Birstler J., Brown R.L. (2019). Addressing adverse childhood experiences and health risk behaviors among low-income, Black primary care patients: testing feasibility of a motivation-based intervention. Gen Hosp Psychiatry.

[78] Selvaraj K., Korpics J., Osta A.D., Hirshfield L.E., Crowley-Matoka M., Bayldon B.W. (2022). Parent perspectives on adverse childhood experiences & unmet social needs screening in the medical home: a qualitative study. Acad Pediatr.

[79] Hosdurga S., Finlay F. (2010). Child protection experience and training: a regional study of international junior paediatricians. Child Abuse Rev.

[80] Candler T.P., Gannon H., Wachira J. (2014). Child protection in a low resource setting; experiences from Paediatric professionals in Kenya. Arch Dis Child.

[81] van den Akker M., Mol S.S., Metsemakers J.F., Dinant G.J., Knottnerus J.A. (2001). Barriers in the care of patients who have experienced a traumatic event: the perspective of general practice. Fam Pract.

[82] Cruz T.H., Woelk L., Vitanzos Cervantes I.C., Kaminsky A. (2023). Barriers to and systems solutions for increasing early childhood home visiting referrals by health care providers serving urban and rural communities. Fam Community Health.

[83] Kuruppu J., Humphreys C., McKibbin G., Hegarty K. (2022). Tensions in the therapeutic relationship: emotional labour in the response to child abuse and neglect in primary healthcare. BMC Prim Care.

[84] Hoffmann Merrild C., Kjeldsen H.C., Milidou I. (2023). Management of child maltreatment suspicions in general practice: a mixed methods study. Scand J Prim Health Care.

[85] Dubowitz H., Lane W.G., Semiatin J.N., Magder L.S. (2012). The SEEK model of pediatric primary care: can child maltreatment be prevented in a low-risk population?. Acad Pediatr.

[86] Dubowitz H., Feigelman S., Lane W., Kim J. (2009). Pediatric primary care to help prevent child maltreatment: the Safe Environment for Every Kid (SEEK) Model. Pediatrics.

[87] Strait J., Meagher S. (2020). Trauma-informed care in pediatrics: a developmental perspective in twelve cases with narratives. Perm J.

[88] Gaba H., Shamaskin-Garroway A.M., Pierson W.E., Berliant M.N. (2022). Racial and ethnic differences in patient-reported acceptability of adverse childhood experience (ACE) screening in adult primary care. J Racial Ethn Health Disparities.

[89] Negriff S., DiGangi M.J., Sidell M., Liu J., Coleman K.J. (2022). Assessment of screening for adverse childhood experiences and receipt of behavioral health services among children and adolescents. JAMA Netw Open.

[90] Woods-Jaeger B., Thompson J.E., Foye-Fletcher A. (2020). Parent engagement in an integrated care parenting intervention to prevent toxic stress. Clin Pract Pediatr Psychol.

[91] Canada R.E., DiRocco D., Day S. (2014). A better approach to opioid prescribing in primary care. J Fam Pract.

[92] Jeffrey J., Do M.C.T., Hajal N. (2021). Using web-based technology to improve depression screening in primary care settings. BMJ Open Qual.

[93] Hartveit M., Vanhaecht K., Thorsen O., Biringer E., Haug K., Aslaksen A. (2017). Quality indicators for the referral process from primary to specialised mental health care: an explorative study in accordance with the RAND appropriateness method. BMC Health Serv Res.

[94] Powell B.J., Proctor E.K., Glass J.E. (2014). A systematic review of strategies for implementing empirically supported mental health interventions. Res Soc Work Pract.

[95] Liness S., Beale S., Lea S., Byrne S., Hirsch C.R., Clark D.M. (2019). The sustained effects of CBT training on therapist competence and patient outcomes. Cogn Ther Res.

[96] Fram M.S., Frongillo E.A., Draper C.L., Fishbein E.M. (2013). Development and validation of a child report assessment of child food insecurity and comparison to parent report assessment. J Hunger Environ Nutr.

[97] Tsipa A., O'Connor D.B., Branley-Bell D. (2021). Promoting colorectal cancer screening: a systematic review and meta-analysis of randomised controlled trials of interventions to increase uptake. Health Psychol Rev.

[98] Agide F.D., Sadeghi R., Garmaroudi G., Tigabu B.M. (2018). A systematic review of health promotion interventions to increase breast cancer screening uptake: from the last 12 years. Eur J Public Health.

[99] Costantini L., Pasquarella C., Odone A. (2021). Screening for depression in primary care with Patient Health Questionnaire-9 (PHQ-9): a systematic review. J Affect Disord.

[100] Flaherty E.G., Jones R., Sege R., Child Abuse Recognition Experience Study Research Group (2004). Telling their stories: primary care practitioners' experience evaluating and reporting injuries caused by child abuse. Child Abuse Negl.

[101] Chappell K.K., Hein L.C., Andrews J.O. (2021). Can we ask everyone? Addressing sexual abuse in primary care. J Nurse Pract.

[102] Forrest C.B., Shadmi E., Nutting P.A., Starfield B. (2007). Specialty referral completion among primary care patients: results from the ASPN referral study. Ann Fam Med.

[103] Patel M.P., Schettini P., O'Leary C.P., Bosworth H.B., Anderson J.B., Shah K.P. (2018). Closing the referral loop: an analysis of primary care referrals to specialists in a large health system. J Gen Intern Med.

[104] Horevitz E., Organista K.C., Arean P.A. (2015). Depression treatment uptake in integrated primary care: how a “warm handoff” and other factors affect decision making by latinos. Psychiatr Serv.

