# Supplementary material for: The effect of adverse childhood experience training, screening, and response in primary care: a systematic review
Source: eClinicalMedicine. 2023 Oct 24;65:102282. doi: 10.1016/j.eclinm.2023.102282 (PMC10725064; doi:10.1016/j.eclinm.2023.102282)
Supplement: Appendix [file mmc1.docx]

**The Effect of Adverse Childhood Experience Training, Screening, and Response in Primary Care: A Systematic Review**

**Online Supplement**

**Appendix 1: Search Study Specification**

**PubMed**English Language; January 1, 1998-May 28, 2023
Search Executed 28 June 2023

"primary care"[tiab] OR "primary doctor"[tiab] OR "primary provider*"[tiab] OR "primary clinic"[tiab] OR "primary healthcare"[tiab] OR "primary health care"[tiab] OR "ambulatory care"[tiab] OR PHC[tiab] OR "general practitioner*"[tiab] OR GP[tiab] OR "general practice"[tiab] OR internist*[tiab] OR "family medicine"[tiab] OR "family practice"[tiab] OR "family doctor*"[tiab] OR "internal medicine"[tiab] OR geriatric*[tiab] OR gerontol*[tiab] OR pediatric*[tiab] OR "community health center*"[tiab] OR "federally qualified health center*"[tiab] OR FQHC*[tiab] OR CBOC*[tiab] OR "community based"[tiab] OR obstetric*[tiab] OR gynecolog*[tiab] OR OBGYN*[tiab] OR "safety net*"[tiab] OR "Primary Health Care"[Mesh:NoExp] OR "Physicians, Primary Care"[Mesh] OR "General Practice"[Mesh] OR "General Practitioners"[Mesh] OR "Geriatrics"[Mesh] OR "Geriatricians"[Mesh] OR "Internal Medicine"[Mesh:NoExp] OR "Family Practice"[Mesh] OR "Pediatrics"[Mesh] OR "Pediatricians"[Mesh] OR "Gynecology"[Mesh] OR "Obstetrics"[Mesh]

AND

"adverse childhood experience*"[tiab] OR "adverse childhood event*"[tiab] OR "childhood adversity"[tiab] OR "childhood trauma"[tiab] "child abuse"[tiab] OR "child neglect"[tiab] OR "child maltreatment"[tiab] OR PEARLS*[tiab] OR "childhood stress*"[tiab] OR "early adversit*"[tiab] OR ACES[tiab] OR "family stress*"[tiab] OR "intergenerational trauma*"[tiab] OR "two generation*"[tiab] OR "Child Abuse"[Mesh] OR "Battered Child Syndrome"[Mesh] OR "Adverse Childhood Experiences"[MeSH] OR "Adult Survivors of Child Adverse Events"[Mesh] OR (("toxic stress"[tiab] OR "stress response*"[tiab] OR "ACE-associated health"[tiab]) AND (child*[tiab] OR "elementary school"[tiab] OR "primary school*"[tiab] OR "middle school*"[tiab] OR "high school*"[tiab] OR preschool*[tiab] OR kindergarten*[tiab] OR youth*[tiab] OR teenager*[tiab] OR kid[tiab] OR kids[tiab] OR adolescen*[tiab] OR "Child"[Mesh] OR "Adolescent"[Mesh]))

**Results: 4,131**

**Web of Science***Science Citation Index Expanded (SCI-EXPANDED), Social Sciences Citation Index (SSCI), Arts & Humanities Citation Index (A&HCI), Book Citation Index – Science (BKCI-S), Book Citation Index – Social Sciences & Humanities (BKCI-SSH), Emerging Sources Citation Index (ESCI)*
English Language; January 1, 1998-May 28, 2023; Article, Review Articles, Early Access, Book Chapters
Search Executed 28 June 2023

TI=("primary care" OR "primary doctor" OR "primary provider*" OR "primary clinic" OR "primary healthcare" OR "primary health care" OR "ambulatory care" OR PHC OR "general practitioner*" OR GP OR "general practice" OR internist* OR "family medicine" OR "family practice" OR "family doctor*" OR "internal medicine" OR geriatric* OR gerontol* OR pediatric* OR "community health center*" OR "federally qualified health center*" OR FQHC* OR CBOC* OR "community based" OR obstetric* OR gynecolog* OR OBGYN* OR "safety net*") OR AB=("primary care" OR "primary doctor" OR "primary provider*" OR "primary clinic" OR "primary healthcare" OR "primary health care" OR "ambulatory care" OR PHC OR "general practitioner*" OR GP OR "general practice" OR internist* OR "family medicine" OR "family practice" OR "family doctor*" OR "internal medicine" OR geriatric* OR gerontol* OR pediatric* OR "community health center*" OR "federally qualified health center*" OR FQHC* OR CBOC* OR "community based" OR obstetric* OR gynecolog* OR OBGYN* OR "safety net*")

AND

TI=("adverse childhood experience*" OR "adverse childhood event*" OR "childhood adversity" OR "childhood trauma" "child abuse" OR "child neglect" OR "child maltreatment" OR PEARLS* OR "childhood stress*" OR "early adversit*" OR "ACES" OR "family stress*" OR "intergenerational trauma*" OR "two generation*") OR AB=("adverse childhood experience*" OR "adverse childhood event*" OR "childhood adversity" OR "childhood trauma" "child abuse" OR "child neglect" OR "child maltreatment" OR PEARLS* OR "childhood stress*" OR "early adversit*" OR "ACES" OR "family stress*" OR "intergenerational trauma*" OR "two generation*") OR (TI=("toxic stress" OR "stress response*" OR "ACE-associated health") AND TI=(child* OR "elementary school" OR "primary school*" OR "middle school*" OR "high school*" OR preschool* OR kindergarten* OR youth* OR teenager* OR "kid" OR "kids" OR adolescen*)) OR (TI=("toxic stress" OR "stress response*" OR "ACE-associated health") AND AB=(child* OR "elementary school" OR "primary school*" OR "middle school*" OR "high school*" OR preschool* OR kindergarten* OR youth* OR teenager* OR "kid" OR "kids" OR adolescen*)) OR (AB=("toxic stress" OR "stress response*" OR "ACE-associated health") AND AB=(child* OR "elementary school" OR "primary school*" OR "middle school*" OR "high school*" OR preschool* OR kindergarten* OR youth* OR teenager* OR kid OR kids OR adolescen*)) OR (AB=("toxic stress" OR "stress response*" OR "ACE-associated health") AND TI=(child* OR "elementary school" OR "primary school*" OR "middle school*" OR "high school*" OR preschool* OR kindergarten* OR youth* OR teenager* OR kid OR kids OR adolescen*))

**Results: 1,657– duplicates = 359**

**CINAHL***exclude Medline*
English Language; January 1, 1998-May 28, 2023; Academic Journals
Search Executed 28 June 2023

TI=("primary care" OR "primary doctor" OR "primary provider*" OR "primary clinic" OR "primary healthcare" OR "primary health care" OR "ambulatory care" OR PHC OR "general practitioner*" OR GP OR "general practice" OR internist* OR "family medicine" OR "family practice" OR "family doctor*" OR "internal medicine" OR geriatric* OR gerontol* OR pediatric* OR "community health center*" OR "federally qualified health center*" OR FQHC* OR CBOC* OR "community based" OR obstetric* OR gynecolog* OR OBGYN* OR "safety net*") OR AB=("primary care" OR "primary doctor" OR "primary provider*" OR "primary clinic" OR "primary healthcare" OR "primary health care" OR "ambulatory care" OR PHC OR "general practitioner*" OR GP OR "general practice" OR internist* OR "family medicine" OR "family practice" OR "family doctor*" OR "internal medicine" OR geriatric* OR gerontol* OR pediatric* OR "community health center*" OR "federally qualified health center*" OR FQHC* OR CBOC* OR "community based" OR obstetric* OR gynecolog* OR OBGYN* OR "safety net*") OR (MH "Primary Health Care") OR (MH "Physicians, Family") OR (MH "Family Practice") OR (MH "Geriatrics") OR (MH "Geriatricians") OR (MH "Internal Medicine") OR (MH "Pediatrics") OR (MH "Pediatricians") OR (MH "Gynecology") OR (MH "Obstetrics")

AND

TI=("adverse childhood experience*" OR "adverse childhood event*" OR "childhood adversity" OR "childhood trauma" "child abuse" OR "child neglect" OR "child maltreatment" OR PEARLS* OR "childhood stress*" OR "early adversit*" OR ACES OR "family stress*" OR "intergenerational trauma*" OR "two generation*") OR AB=("adverse childhood experience*" OR "adverse childhood event*" OR "childhood adversity" OR "childhood trauma" "child abuse" OR "child neglect" OR "child maltreatment" OR PEARLS* OR "childhood stress*" OR "early adversit*" OR ACES OR "family stress*" OR "intergenerational trauma*" OR "two generation*") OR (MH "Child Abuse+") OR (MH "Adverse Childhood Experiences") OR (MH "Child Abuse Survivors") OR (TI=("toxic stress" OR "stress response*" OR "ACE-associated health") AND (TI=(child* OR "elementary school" OR "primary school*" OR "middle school*" OR "high school*" OR preschool* OR kindergarten* OR youth* OR teenager* OR kid OR kids OR adolescen*) OR (MH "Child+") OR (MH "Adolescence+"))) OR (TI=("toxic stress" OR "stress response*" OR "ACE-associated health") AND (AB=(child* OR "elementary school" OR "primary school*" OR "middle school*" OR "high school*" OR preschool* OR kindergarten* OR youth* OR teenager* OR kid OR kids OR adolescen*) OR (MH "Child+") OR (MH "Adolescence+"))) OR (AB=("toxic stress" OR "stress response*" OR "ACE-associated health") AND (AB=(child* OR "elementary school" OR "primary school*" OR "middle school*" OR "high school*" OR preschool* OR kindergarten* OR youth* OR teenager* OR kid OR kids OR adolescen*) OR (MH "Child+") OR (MH "Adolescence+"))) OR (AB=("toxic stress" OR "stress response*" OR "ACE-associated health") AND (TI=(child* OR "elementary school" OR "primary school*" OR "middle school*" OR "high school*" OR preschool* OR kindergarten* OR youth* OR teenager* OR kid OR kids OR adolescen*) OR (MH "Child+") OR (MH "Adolescence+")))

**Results:** **437 – duplicates = 234**

**APA PsycInfo**

English Language; January 1, 1998-May 28, 2023; Academic Journals; Books
Search Executed 28 June 2023

TI=("primary care" OR "primary doctor" OR "primary provider*" OR "primary clinic" OR "primary healthcare" OR "primary health care" OR "ambulatory care" OR PHC OR "general practitioner*" OR GP OR "general practice" OR internist* OR "family medicine" OR "family practice" OR "family doctor*" OR "internal medicine" OR geriatric* OR gerontol* OR pediatric* OR "community health center*" OR "federally qualified health center*" OR FQHC* OR CBOC* OR "community based" OR obstetric* OR gynecolog* OR OBGYN* OR "safety net*") OR AB=("primary care" OR "primary doctor" OR "primary provider*" OR "primary clinic" OR "primary healthcare" OR "primary health care" OR "ambulatory care" OR PHC OR "general practitioner*" OR GP OR "general practice" OR internist* OR "family medicine" OR "family practice" OR "family doctor*" OR "internal medicine" OR geriatric* OR gerontol* OR pediatric* OR "community health center*" OR "federally qualified health center*" OR FQHC* OR CBOC* OR "community based" OR obstetric* OR gynecolog* OR OBGYN* OR "safety net*") OR (DE "Primary Health Care") OR (DE "Geriatrics") OR (DE "Gerontology") OR (DE "Pediatrics") OR (DE "Gynecology") OR (DE "Obstetrics")

AND

TI=("adverse childhood experience*" OR "adverse childhood event*" OR "childhood adversity" OR "childhood trauma" "child abuse" OR "child neglect" OR "child maltreatment" OR PEARLS* OR "childhood stress*" OR "early adversit*" OR ACES OR "family stress*" OR "intergenerational trauma*" OR "two generation*") OR AB=("adverse childhood experience*" OR "adverse childhood event*" OR "childhood adversity" OR "childhood trauma" "child abuse" OR "child neglect" OR "child maltreatment" OR PEARLS* OR "childhood stress*" OR "early adversit*" OR ACES OR "family stress*" OR "intergenerational trauma*" OR "two generation*") OR (DE "Child Abuse") OR (DE "Battered Child Syndrome") OR (DE "Childhood Adversity") OR (TI=("toxic stress" OR "stress response*" OR "ACE-associated health") AND TI=(child* OR "elementary school" OR "primary school*" OR "middle school*" OR "high school*" OR preschool* OR kindergarten* OR youth* OR teenager* OR kid OR kids OR adolescen*)) OR (TI=("toxic stress" OR "stress response*" OR "ACE-associated health") AND AB=(child* OR "elementary school" OR "primary school*" OR "middle school*" OR "high school*" OR preschool* OR kindergarten* OR youth* OR teenager* OR kid OR kids OR adolescen*)) OR (AB=("toxic stress" OR "stress response*" OR "ACE-associated health") AND TI=(child* OR "elementary school" OR "primary school*" OR "middle school*" OR "high school*" OR preschool* OR kindergarten* OR youth* OR teenager* OR kid OR kids OR adolescen*)) OR (AB=("toxic stress" OR "stress response*" OR "ACE-associated health") AND AB=(child* OR "elementary school" OR "primary school*" OR "middle school*" OR "high school*" OR preschool* OR kindergarten* OR youth* OR teenager* OR kid OR kids OR adolescen*))

**Results: 630 – duplicates = 251**

| **Appendix 2. Included Studies**  **A. Provider-Related Outcomes – Pediatric Patients** | | | | | | | | | |  |
| --- | --- | --- | --- | --- | --- | --- | --- | --- | --- | --- |
| Authorship | Adversity Focus | Intervention Focus | Screening Instrument | Setting | Study Period | Analytic  Sample Size | Study Design | Strength of Evidence | Provider-Related Outcomes | |
| Abatemarco et al., 2008 (69) | Child abuse and neglect, including issues related to coping with crying, discipline, safety, and toilet training | Screening; response | *Practicing Safety* Questionnaire | NJ, USA | 2003-2006 | 3 primary care practices | Prospective cohort, no comparison group | Very low (+) | 1. Referrals: Clinics reported an increase in referrals or connections to additional resources. One site increased referrals to social workers, one increased interactions with health educators, and one increased use of triage nurses for supportive services (percent increase not reported). 2. Screening Behavior: All three clinics increased rates of screening, specifically maternal depression and psychosocial assessments, with two sites integrating screening into routine care (percent increase not reported). 3. Clinical Care: Two of three clinics posted community resource materials, and one of three clinics had patient education information readily available. | |
| Abatemarco et al., 2018 (68) | Child maltreatment related to maternal depression, attachment, bonding, coping with crying, discipline, and toilet training | Screening; response | *Practicing Safety* Questionnaire | USA | 2011 | 14 clinics and associated core improvement teams; 16 provider key informants | Interrupted time series; semi-structured interviews with providers | Very low (+) | 1. Reporting Practices: Documentation practices for patient needs and concerns, such as safety concerns and needs related to discipline and toilet training, increased significantly after the intervention (p=0.02). 2. Referrals: In interviews, providers reported working more closely with clinical social workers to improve communication and increase referrals to community resources. 3. Screening Behavior: The intervention resulted in increased screening rates for ACEs (p=0.04). 4. Clinical Care: Providers were significantly more likely to provide evidence-based anticipatory guidance, both written and verbal, to patients regarding responses to crying, maternal depression, discipline, and toilet training after the intervention (p=0.01). | |
| Bannon et al., 2001 (49) | Child abuse and neglect | Training | Not Reported | London, UK | 1999 | 31 general practitioner registrars | Prospective cohort, no comparison group | Very low (+) | 1. Knowledge: Providers' knowledge of legal issues surrounding child protection increased significantly following the new training package: from a pre-training mean score of 1.9 to a post-training mean score of 3.5 on a five-point scale (p<0.01). 2. Confidence/Self-Efficacy: Provider confidence in assessing and responding to child protection needs increased significantly from a pre-training mean score of 2.1 versus a post-training mean score of 3.7 on a five-point scale (p<0.05). 3. Reporting Practices: Providers' understanding of their reporting responsibilities for CPS increased significantly from a pre-training mean score of 2.5 versus a post-training mean score of 4.0 on a five-point scale (p<0.01). | |
| Berg-Poppe et al., 2022 (47) | Traumatic stress; historical trauma | Training | Not Reported | Midwest, USA | Not listed | 13 pediatric health care providers | Prospective cohort, no comparison group | Low (++) | 1. Knowledge: Knowledge about beliefs related to trauma increased post-intervention (score: 4.47 before vs. 4.96 after, p<0.01), and foundational knowledge about trauma-informed care also increased (score: 2.46 before vs. 3.42 after, p<0.01). Providers with more training reported higher levels of knowledge (p<0.01). 2. Confidence/Self-Efficacy: Increase in reported self-efficacy from before to after the intervention (score: 4.37 before vs. 4.82 after, p<0.05). Providers with more face-to-face training reported higher levels of self-efficacy (p<0.01). | |
| Bodendorfer et al., 2020 (65) | Adverse childhood experiences (omnibus) | Screening | Not Reported | Midwest, USA | 2017-2018 | 13 primary care providers; 238 parents/guardians | Repeated cross-sectional, survey | Low (++) | 1. Confidence/Self-Efficacy: No changes were observed over time in feeling prepared and comfortable discussing ACEs (p>0.05); the seven providers who completed the quarterly surveys already felt prepared and comfortable. 2. Rapport with Patients: Most providers (71%) reported that parents/guardians were receptive to the discussion on ACEs. 3. Reporting Practices: Only 9% of discussions led to disclosure of an ACE. No discussions led to reporting of child abuse. | |
| Bryant et al., 2020 (43) | Adverse childhood experiences (omnibus) | Training; screening | Center for Youth & Wellness (CYW) ACEs Screening Tool | Mid-Atlantic, USA (urban) | Not listed | 59 providers | Prospective cohort, no comparison group | Low (++) | 1. Knowledge: Providers' knowledge of ACEs and screening increased from a mean score of 3.5 to 4.3 on a five-point Likert scale (p<0.001). 2. Confidence/Self-Efficacy: Providers' comfort in screening for ACEs increased from a mean score of 3.1 to 3.8 on a five -point Likert scale (p<0.001). 3. Screening Behavior: A large majority (93%) of providers implemented the screening for ACEs, with 87% reporting that they agreed or strongly agreed with continuing to screen. | |
| Brennan et al., 2022 (70) | Social determinants of health, socioemotional development, and perinatal depression | Screening; response | Hunger Vital Signs; American Academy of Family Physicians (AAFP) Screening Tool | Indiana, USA | 2020-2021 | 10 practices | Repeated cross-sectional, non-survey | Very low (+) | 1. Referrals: Increase in needed referrals fulfilled from 37% to 57% (p = 0.003). No significant improvement in referring patients who screened positive (81% vs 89%, p = 0.0949). 2. Screening Behavior: SDOH screenings increased significantly from 21% to 62% (p < 0.0002). | |
| Campbell et al., 2020 (60) | Child maltreatment; toxic stress | Training | Safe Environment for Every Kid (*SEEK*) Study Questionnaire | UT, USA | 2014-2015 | 5 health care professionals; 8 CPS staff | Focus group discussions with providers | Very low (+) | 1. Reporting Practices: In focus group discussions, providers reported that the social risk screening process helped foster a collaborative relationship between families and clinics for referrals to CPS. | |
| Candler et al., 2014 (80) | Child abuse | Response | Not Reported | Kenya | Not listed | 58 pediatric professionals | Cross-sectional, survey | Very low (+) | 1. Knowledge: 29% of pediatric professionals received formal training for child protection. Those who completed training reported that it was useful (mean=7.2 out of 10). 2. Confidence/Self-Efficacy: Pediatric professionals were most confident assessing physical abuse (mean=6.6 out of 10) and neglect (mean=6.6) and least confident assessing emotional abuse (mean=4.8) and factitious illness (mean=5.3). 3. Reporting Practices: 85% of pediatric professionals reported less than five child protection cases per year, 64% understood their local child protection reporting policies, and 22% knew how to access national guidelines. | |
| Chokshi et al., 2020 (48) | Adverse childhood experiences (omnibus) | Training | Not Reported | Washington DC, USA | Not listed | 35 providers | Cross-sectional, survey | Very low (+) | 1. Knowledge: The post-intervention survey found that the physicians observed a significant increase in their knowledge of ACEs (4.4 vs. 2.5, p<0.001). 2. Confidence/Self-Efficacy: The intervention increased the confidence that physicians had in screening for ACEs (4.3 vs. 2.7, p<0.001). 3. Screening Behavior: In a post-session open-ended questionnaire, physicians indicated that they would be more likely to integrate ACEs screening into their work and use a core component of the training (the 7 C’s) to build resilience in their patients. | |
| Chung et al., 2019 (68) | Child abuse and neglect | Screening | *Practicing Safety*  Questionnaire | Philadelphia, PA, USA | 2013-2015 | 581 patient medical charts | Prospective cohort, no comparison group | Moderate (+++) | 1. Screening Behavior: Screening for infant crying increased from 0% to 83% (p<0.001). Screening for maternal depression increased from 0% to 54% (p<0.001). Screening for discipline, temper tantrums, and toilet training increased from 0% to 65%, 6% to 72%, and 36% to 82%, respectively (each p<0.001). 2. Clinical Care: At post-intervention, providers gave patients more resources related to maternal depression (0% pre-intervention vs. 58% post-intervention, p<0.001) and discipline practices (0% pre-intervention vs. 61% post-intervention, p<0.001). | |
| Crenshaw et al., 2021 (67) | Adverse childhood experiences (omnibus) | Screening | CYW-ACEs Screening Tool | Not listed | 2018-2020 | 8 physicians; 232 families | Cross-sectional, survey | Very low (+) | 1. Referrals: 6% of those screened were referred to additional resources. Of those who reported four or more ACEs, 16% were deemed to warrant for a referral. Only 3% of those who had less than four ACEs were referred to resources. 2. Screening Behavior: Between 58% to 100% of screenings were completed across implementation phases, with 20% being incomplete upon starting. The reasons for incomplete screenings included staff forgetting to deliver the screen, physicians failing to document screening activities, caregivers misunderstanding instructions, misplaced screening forms, and caregiver refusals. | |
| Cruz et al., 2023 (82) | Adverse childhood experiences, socioeconomic disadvantage | Response | Not Reported | New Mexico, USA | 2016-2019 | 37 providers | Semi-structured interviews with providers | Very low (+) | 1. Knowledge: Lack of knowledge was mentioned as a barrier for all participants, though where the lack of knowledge was varied. Providers in urban areas were aware of the local and tribal ECHV programs. Providers outside urban areas did not know which ECHV programs were available in their community. 2. Confidence/Self-Efficacy: There was a lack of trust in ECHV programs. Providers were concerned about referring a patient to an unknown entity. 3. Rapport: Providers were concerned that a poor experience with a home-visiting program could negatively affect the provider-patient relationship. | |
| Dara et al., 2013 (53) | Child abuse and neglect | Training | Not Reported | USA | 2009 | 119 pediatric chief residents | Cross-sectional, survey | Very low (+) | 1. Knowledge: More training was correlated with respondents’ confidence in identifying child abuse (p=0.03), conducting a child abuse and neglect history (p=0.01), and awareness of appropriate ethnic and cultural practices (p=0.01). | |
| DiGangi and Negriff, 2020 (31) | Adverse childhood experiences (omnibus) | Screening | Kaiser Permanente–Centers for Disease Control and Prevention (KP-CDC) Questionnaire | Los Angeles and Orange Counties, CA, USA | 2018-2019 | 7,056 children, ages 3 to 13 | Repeated cross-sectional, survey | Very low (+) | 1. Screening Behavior: Screening rates varied by location, ranging from 48% to 73%. 7,056 screenings were started, consisting of 99% of all screenings, with only seven screenings that were refused. 53% of the target population for 3- and 5-year-olds, 37% of 10-year-olds, and 32% of 13-year-olds participated in screening. | |
| DiGiovanni et al., 2023 (59) | Pediatric traumatic experiences | Training; screening | UCLA Abbreviated Post Traumatic Stress Disorder Reaction Index (aPTSDRI) | Portland, ME, USA | 2016-2019 | 15,175 well-child visits | Cross-Sectional, non-survey | Low (++) | 1. Referrals: Among a sample of 124 children who screened positive for PTSD and were not already connected to behavioral health services, 46.5% were referred to services. 2. Screening Behavior: During the first phase of the intervention, the two-question screening completion rate was 49.6% of well-child visits. By the third phase, the completion rate rose to 89.8%. Between the first and second phase, three sites transitioned from a verbal to a written questionnaire and saw a significant increase in positive screens (p<0.0001), whereas the two sites that used the written questionnaire throughout the project did not see an increase. | |
| Eismann et al., 2019 (51) | Child maltreatment, including parental depression, substance use, and stress, domestic violence, discipline, and food insecurity | Training; screening; response | *SEEK* Study Questionnaire | Not listed (urban, suburban, and rural) | 2015-2016 | 16 providers; 1,190 targeted families | Cross-sectional, survey; semi-structured interviews with providers | Low (++) | 1. Knowledge: In interviews, providers expressed that the program helped to increase their knowledge of local social services and resources for their patients, though a recurring refrain among providers pertained to incomplete resource knowledge for referrals. 2. Confidence/Self-Efficacy: In interviews, providers expressed that they did not know local resources well enough to feel comfortable referring their patients. They also shared concerns that families may feel uncomfortable talking with social workers who were not affiliated with their organization. 3. Screening Behavior: Across the three settings, 75% to 93% of targeted patients were screened for ACEs. 4. Clinical Care: Across the three settings, providers conducted motivational interviewing with 61% to 81% of patients identified as in-need of the intervention. | |
| Eismann et al., 2023 (56) | Childhood adversity and toxic stress | Training; screening; response | *SEEK* Study Questionnaire | Midwest, USA | 2017-2019 | 27,419 pediatric patients | Randomized controlled trial | High (++++) | 1. Referrals: During the SEEK intervention, patients were more likely to receive any type of referral (OR=1.45) and more likely to receive a behavioral health referral (OR=1.54). | |
| Feigelman et al., 2011 (45) | Psychosocial risk factors, including parental depression, substance use, and stress, domestic violence, discipline, and food insecurity | Training | *SEEK* Study Questionnaire | Not listed (urban) | Not listed | 95 residents; 558 families | Randomized controlled trial | Moderate (+++) | 1. Knowledge & Confidence/Self-Efficacy: After training, those in the intervention group improved more than the control group on self-assessed knowledge and confidence identifying depression (p<0.01), IPV (p=0.03), and stress (p=0.04) among patients, with some indication that differences were sustained through 18 months. 2. Screening Behavior: After training, intervention group providers were more likely to screen for ACEs than those in the control group, particularly screening for depression (88% vs. 16%, p<0.001). | |
| Froula et al., 2017 (46) | Child abuse; toxic stress | Training; screening | Not Reported | Rochester, NY, USA | Not listed | 34 resident physicians | Prospective cohort, no comparison group | Low (++) | 1. Knowledge: Residents were better able to identify toxic stress-related complaints after the intervention (2.68 pre-score average vs. 2.47 post-score average, p=0.04), and this was sustained three months later (p=0.03). 2. Clinical Care: Residents reported they would be more likely to integrate conversations about risk factors during an assessment, but the effect was non-significant (37% vs. 29%, p>0.05). However, residents were more likely to report being able to provide interpersonal support and resources when discussing potential stress issues (37% vs. 6%, p=0.006), and this increased in the three-month follow-up period (41% vs. 37%, p=0.02). | |
| Garg et al., 2007 (58) | Psychosocial risk factors, including parental unemployment, lack of education, substance use and depression, domestic violence, homelessness, and food insecurity | Training; screening; response | Well Childcare Visit, Evaluation, Community Resources, Advocacy, Referral, Education (WE CARE) Survey | Not listed | 2006 | 45 residents; 200 parents | Randomized controlled trial | High (++++) | 1. Referrals: Compared to those in the control group, parents in the intervention group were significantly more likely to receive a referral from providers (adjusted OR=6.7, p<0.001). 2. Screening Behavior: Compared to those in the control group, parents in the intervention group discussed more psychosocial topics during well-child check-up visits (2.9 vs 1.8, p<0.01). | |
| Gerlach et al., 2021 (73) | Adverse childhood experiences (omnibus) | Screening; response | Not Reported | Central TX, USA | 2016 | 114 providers | Cross-sectional, survey | Low (++) | 1. Screening Behavior: 28.4% of providers did not screen for ACEs. Most commonly, providers (51.4%) screened for anyone in the home having a mental illness or substance use disorder. Least frequently screened ACEs were ethnic/racial discrimination (6.5%) and community violence (9.4%). 2. Clinical Care: The most common strategies to deal with ACEs were using on-site clinical staff and resources, including social workers or therapists (76.4%) or materials/publications (71.9%). In multivariate analysis, a one-unit increase in self-reported ACE knowledge increased the odds of implementing the strategies of identifying family strengths by 2.00 (p<0.05) and implementing other interventions by 1.83 (p<0.05). | |
| Henry et al., 2003 (50) | Child abuse and neglect | Training | Not Reported | Japan | Not listed | 156 nurse educators, clinicians, and managers | Cross-sectional, survey | Very low (+) | 1. Knowledge: Providers reported strongly agreeing with a majority of questions pertaining to knowledge about actions in response to suspected child abuse. Open-ended survey responses demonstrated satisfaction with the program and with having gained new knowledge. | |
| Hoffmann Merrild et al., 2023 (84) | Child maltreatment | Response | Not Reported | Denmark | 2019-2020 | 1,252 survey respondents; 20 interviewees | Cross-sectional, survey; semi-structured interviews with providers | Very low (+) | 1. Reporting Practices: When reporting or discussing suspected child abuse and/or neglect, providers preferred to discuss with social services (94%), a colleague (63%), or the caregiver (60%). Barriers to reporting included fear of consequences for the child and continued abuse/neglect, ambiguity with the accurate diagnosis, and involvement of family. | |
| Hosdurga and Finlay, 2010 (79) | Child abuse and neglect | Response | Not Reported | Southwest England, UK | Not listed | 50 pediatric junior doctors | Cross-sectional, survey | Very low (+) | 1. Knowledge: About three-quarters (73%) of doctors from nine different geographies had no undergraduate training in child protection. 27% had undergraduate training through lectures and clinical observations. Of those with overseas postgraduate education, 68% had no training and 16% had some training. Working at hospitals in the UK, 95% received child protection training. | |
| Jee et al., 2020 (55) | Adverse childhood experiences (omnibus) | Training | American Academy of Pediatrics (AAP) Trauma Toolkit | Rochester, NY, USA | 2017-2018 | 52 survey respondents; 16 interviewees; 36 focus group participants | Cross-sectional, survey; focus group discussions with providers | Very Low (+) | 1. Confidence/Self-Efficacy: Prior to training, a majority of survey respondents (62%) expressed they did not feel confident managing trauma in their pediatric office. After the training, providers shared in focus group discussions that they felt limited in their capacity to help families in difficult situations. | |
| Khan et al., 2005 (52) | Child abuse and maltreatment | Training | Not Reported | NY, USA | Not listed | 424 physicians | Cross-sectional, survey | Very low (+) | 1. Knowledge: A large majority of participants (88%) agreed that the course made an impact on their knowledge of and ability to recognize child abuse cases. This did not vary significantly as a function of when the course was taken (p>0.05). 2. Reporting Practices: Taking the training course did not impact reporting of child abuse cases (p>0.05). | |
| Kia-Keating et al., 2019 (26) | Adverse childhood experiences (omnibus); adverse community experiences, including discrimination, violence, and natural disaster | Screening | Adaptation of CYW-ACEs Screening Tool | Not listed | Not listed | 164 patients and caregivers | Cross-sectional, survey; semi-structured interviews with providers | Very low (+) | 1. Knowledge: In interviews, providers shared a desire for ongoing training regarding ACEs screening and response, including to address staff turnover and to emphasize best practices for introducing and discussing ACEs. 2. Confidence/Self-Efficacy: Providers expressed during interviews that they initially felt uncomfortable with the screening, but over time felt more confident and accepting. 3. Rapport with Patients: In interviews, providers shared that they believed ACEs screening helped strengthen patient-provider rapport. 4. Screening Behavior: Most infants and caregivers (92%) were screened for ACEs. | |
| Konijnendijk et al., 2019 (71) | Child abuse and neglect | Screening | Not Reported | Eastern Netherlands | 2014 | 152 providers | Randomized controlled trial | Moderate (+++) | 1. Screening Behavior: No significant difference in adherence to guidelines on child protection was observed between providers using the computer-assisted guidelines and those who did not use the guidelines (p>0.05). Those who used the guidelines spent significantly less time performing guideline-based care (75 min vs. 135 min, p=0.01). | |
| Kuruppu et al., 2022 (83) | Child abuse and neglect | Response | Not Reported | Australia | Not listed | 22 general practitioners and 8 nurses | Semi-structured interviews with providers; focus group discussions with providers | Low (++) | 1. Rapport with Patients: During interviews and focus groups, providers expressed concerns that responding to child abuse could betray the trust of the patient-provider relationship. 2. Reporting Practices: With regard to mandatory reporting laws, providers felt conflicted in having to choose to either support caregivers as their patients who may be engaging in abusive behavior or to protect children experiencing abuse. | |
| Liu et al., 2021 (64) | Adverse childhood experiences (omnibus) | Screening | CYW-ACEs Screening Tool | Not listed (urban) | 2017-2019 | 5 primary care providers; 163 pediatric patients | Prospective cohort, no comparison group; semi-structured interviews with providers | Very low (+) | 1. Confidence/Self-Efficacy: Providers reported in interviews that, through regular assessment of ACEs, they were able to identify information about patients, including details on trauma and safety concerns, that would not have been identified otherwise. 2. Rapport with Patients: During interviews, providers expressed that ACE screening created improvements in child-parent relations and provider-patient relations. 3. Referrals: Providers expressed that, through increased awareness of patients' ACEs, they were able to conduct more informed referrals. 4. Screening Behavior: 97% of eligible study participants completed an ACEs screening. 5. Clinical Care: Providers expressed that, through increased awareness of patients' ACEs, they were able to conduct informed treatment planning. | |
| Lloyd et al., 2021 (54) | Adverse childhood experiences (omnibus) | Training | Not Reported | Baltimore, MD, USA | 2019-2020 | 22 pediatric residents | Prospective cohort, no comparison group | Very low (+) | 1. Confidence/Self-Efficacy: After the training program, pediatric residents reported increased comfort with trauma-informed skills across all six items (p<0.05) from an average of 3.2 (pre-training) to 4.1 (post-training) on a five-point Likert scale. Topics included identifying protective factors, counseling on resilience, and inquiring whether a patient had experienced an ACE. | |
| Marie-Mitchell et al., 2019 (27) | Child abuse and neglect; domestic violence; substance use; mental illness, incarceration | Screening | Whole Child Assessment (WCA) | San Bernardino, CA, USA | 2014-2017 | 1,100 patient medical charts; 30 caregivers | Repeated cross-sectional, non-survey; semi-structured interviews with providers | Low (++) | 1. Reporting Practices: After initiating screening, documentation of ACEs increased significantly among children ages 5-11, from identifying three or more ACEs in 0% of the population (from September 2014 to January 2015) to identifying three or more ACEs in 15-19% of the population (from October 2016 to July 2017). | |
| Marsicek et al., 2019 (28) | Adverse childhood experiences (omnibus); childhood illness; bullying, foster care, or deportation | Screening | CYW-ACEs Screening Tool | St. Petersburg, FL, USA | 2017-2018 | 24 providers; 1,206 children | Cross-sectional, survey | Low (++) | 1. Confidence/Self-Efficacy: Providers experienced a non-significant improvement (p=0.219) in comfort discussing abuse with their patients after the intervention. 2. Screening Behavior: Providers increased ACEs screening from 0% before the intervention to 60% after the intervention. | |
| Popp et al., 2020 (74) | Adverse childhood experiences (omnibus) | Screening | Not Reported | Midwest, USA | Not listed | 48 pediatric and family physicians and physician assistants | Cross-sectional, survey | Very low (+) | 1. Screening Behavior: 47% regularly screened for ACEs. Knowing about research on ACE impacts on health (p<0.01) and training on ACE screening (p<0.01) were associated with screening for ACEs. Lack of education (66%), assessment tools (62%) and too many conditions for which to screen (63%) were the most cited reasons for not screening. | |
| Quizhpi et al., 2019 (75) | Adverse childhood experiences (omnibus) | Screening; response | CYW-ACEs Screening Tool | South Central Appalachia, TN, USA | 2017-2019 | 16 healthcare providers; 51 parents/caregivers | Cross-sectional, survey | Very low (+) | 1. Referrals: Over one-third of families who were screened for ACEs were referred to a positive parenting program, and over half of parents of infants were referred to parenting programs for infants. | |
| Reading et al., 2022 (74) | Adverse childhood experiences (omnibus) | Screening | Childhood Experiences Questionnaire and Pediatric ACEs and Related Life Events Screener (PEARLS) | CA, USA | 2021 | 18 physicians | Semi-structured interviews with providers | Low (++) | 1. Reporting Practices: Providers considered both ACE screening scores and patient symptoms when deciding whether to report an ACE and recommend or initiate interventions. 2. Screening Behavior: Providers used considerably different workflow protocols for screening and response to ACEs. 3. Clinical Care: Providers used different criteria to determine clinical response to ACEs and had no consensus on an ACE score that would require a specific response. | |
| Rosado et al., 2023 (57) | Adverse childhood experiences (omnibus) | Training; screening | ACEs Plus+ | Florida | Not listed | 2,347 children; 6 pediatricians | Cross-sectional, survey; focus group discussions with providers | Very low (+) | 1. Confidence/Self-Efficacy: All six pediatricians reported finding the protocolized screening superior to addressing ACEs on their own. Even with a screener, providers were concerned about false positives. 2. Screening Behavior: Five out of six pediatricians reported that screening interrupted clinical workflow. 3. Clinical Response: Before the program, pediatricians reported being inconsistent in addressing ACEs and there was poor collaboration between team members. | |
| Schmitz et al., 2019 (41) | Adverse childhood experiences (omnibus); toxic stress; resiliency | Training | Not Reported | Not listed | 2016-2018 | 11 pediatric residents | Prospective cohort, no comparison group | Very low (+) | 1. Knowledge: After completing the online module, pediatric residents' self-reported confidence in ACEs knowledge increased from a median of 3 of 5 to 4 of 5 (p<0.05). 2. Confidence/Self-Efficacy: Pediatric residents' self-reported confidence discussing ACEs and toxic stress with patients and parents increased from a median of 2 of 5 to 4 of 5 (p<0.05). 3. Clinical Care: Pediatric residents' self-reported frequency discussing ACEs with patients and parents increased significantly, from 28% to 42% (p<0.01). | |
| Selvaraj et al., 2019 (29) | Adverse childhood experiences (omnibus); unmet needs, including parental unemployment, lack of education, and food and housing insecurity | Screening | Addressing Social Key (ASK) Questions for Health Questionnaire | Chicago, IL, USA | 2016-2017 | 2,569 parents/guardians | Cross-sectional, survey | Low (++) | 1. Referrals: Community referral rates increased from 2.0% to 13.3% (p<0.001) at one of the study sites after screening implementation. | |
| Selvaraj et al., 2022 (78) | Adverse childhood experiences; family unmet social needs | Screening; response | Not Reported | Chicago, IL | 2018-2019 | 40 parents | Semi-structured interviews with parents | Very low (+) | 1. Rapport: To build rapport, parents recommend that providers ask genuinely, foster trust, and explain the process for screening and response. 2. Reporting Practices: Parents are sometimes reluctant to share because of the potential involvement of CPS, fear of deportation, and loss of confidentiality/privacy. | |
| Strait and Meagher, 2020 (87) | Adverse childhood experiences (omnibus) | Screening; response | CYW-ACEs screening tool | Not listed | Not listed | 12 patients | Qualitative | Very low (+) | 1. Screening Behavior: Screening for ACEs and using trauma-informed care allowed providers to start dialogue with families about therapeutic interventions. | |
| van den Akker et al., 2001 (81) | Traumatic events, including accidents, incest, and physical and sexual abuse | Response | Not Reported | Netherlands | Not listed | 211 general practitioners | Cross-sectional, survey; semi-structured interviews with providers | Very low (+) | 1. Knowledge: 20% of practitioners reported that they encounter barriers caring for children who are physically or sexually abused, with the most common barriers being lack of knowledge of signs of abuse (42%) and danger (48%), and insufficient skills to confront parents with suspected abuse (52%) or to start treatment (62%). | |
| Walbeehm-Hol et al., 2022 (63) | Adverse childhood experiences (omnibus) | Screening | Not Reported | Netherlands | 2018-2019 | 548 pediatric health care providers | Cross-sectional, survey | Very low (+) | 1. Knowledge: 66.9% of providers were familiar with the term Adverse Childhood Experiences, 29.6% were familiar with the term toxic stress, 91.6% were familiar with the relationship between multiple ACEs and adverse health. 2. Screening Behavior: 17% reported regularly checking for ACEs, and 18% did not do any ACEs checks. 65% reported occasionally asking about ACEs. 17% asked only the parents, 9.6% asked only the children, and 73.4% asked both the parents and the children. | |
| Wilson et al., 2005 (44) | Child neglect; parental alcoholism | Training | Not Reported | Boston, MA, USA | Not listed | 37 pediatric residents | Prospective cohort, comparison group; focus group discussions with providers | Moderate (+++) | 1. Knowledge: Neither the intervention nor the control group differed on baseline knowledge (p=0.60). Neither groups’ knowledge increased after the intervention (p=0.06 for intervention group, p=0.34 for control group). 2. Confidence/Self-Efficacy: During focus group discussions, pediatric residents reported feeling discouraged and powerless when attempting to address suspected cases of child neglect and parental alcoholism. | |
| Yaun et al., 2022 (76) | Adverse childhood experiences (omnibus); social determinants of health | Screening | PEARLS; Accountable Health Communities (AHC) Health-related Social Needs (HRSN) | Memphis, TN, USA | 2018-2019 | 246 children; 2 outreach coordinators | Prospective cohort, no comparison group | Very low (+) | 1. Referrals: 33.1% of families who were screened by the outreach staff received a referral to further psychological services. Outreach coordinators proactively followed up on referrals (29.4%) and confirmed appointments (18.3%) and well-being check-ins (13.0%). | |
| **B. Provider-Related Outcomes – Adult Patients**   \| Authorship \| Adversity Focus \| Intervention Focus \| Screening Instrument \| Setting \| Study Period \| Analytic  Sample Size \| Study Design \| Strength of Evidence \| Provider-Related Outcomes \| \| --- \| --- \| --- \| --- \| --- \| --- \| --- \| --- \| --- \| --- \| \| Alhoway-mel et al., 2023 (62) \| Childhood abuse, including physical and emotional abuse and neglect \| Screening \| Not Reported \| Riyadh and Madinah, Saudi Arabia \| 2021 \| 126 healthcare providers \| Cross-sectional, survey \| Very low (+) \| 1. Knowledge: Most of the HCPs (69.8%) received no training and 15.1% received school training in screening for childhood abuse. 2. Confidence/Self-Efficacy: 57.9% of HCPs were moderately to very confidently able to screen for childhood abuse. 61.1% were moderate to very confident using information about screening for past adversities to guide care. 3. Rapport: HCPs were uncomfortable sharing inquiries about psychosocial issues (X2(2) = 8.592; p<0.05) with patients, were concerned about offending their patients (X2(2) = 9.531; p<0.01), and had difficulty verifying disclosers and reports (X2(2) = 7.317; p<0.01). 4. Referrals: About half of the HCPs would usually or always suggest a referral to a specialist in mental health when asked about responding to adult discloses childhood abuse (47.2%). 5. Screening Behavior: 15.9% reported that they would usually or always screen new female patients for childhood abuse and 22.2% would screen female patients during follow-up visits. 15.9% screened new male patients and 21.4% screened male patients during the follow-up visit. Overall, 30.2% reported that they would usually or always screen patients for childhood abuse. 6. Clinical Response: 38.4% of HCPs usually or always discuss in detail the history of abuse and the ways to relieve symptoms. \| \| Carroll et al., 2005 (61) \| Child abuse, maternal depression, and family violence \| Training; screening \| Antenatal Psychosocial Health Assessment (ALPHA) \| Ontario, Canada \| Not listed \| 48 providers; 127 patients \| Randomized controlled trial \| Moderate (+++) \| 1. Reporting Practices: The group assigned to use the form did not identify more psychosocial concerns (39% vs. 28%, p=0.14). in the group administering the form, there was a greater frequency of women reporting an experience of and/or witnessing child abuse (OR=7.0, CI=1.3,7.5). \| \| Cruz et al., 2023 (82) \| Adverse childhood experiences, socioeconomic disadvantage \| Response \| Not Reported \| New Mexico, USA \| 2016-2019 \| 37 providers \| Semi-structured interviews with providers \| Very low (+) \| 1. Knowledge: Lack of knowledge was mentioned as a barrier for all participants, though where the lack of knowledge was varied. Providers in urban areas were aware of the local and tribal ECHV programs. Providers outside urban areas did not know which ECHV programs were available in their community. 2. Confidence/Self-Efficacy: There was a lack of trust in ECHV programs. Providers were concerned about referring a patient to an unknown entity. 3. Rapport: Providers were concerned that a poor experience with a home-visiting program could negatively affect the provider-patient relationship. \| \| Dubowitz et al., 2011 (42) \| Child maltreatment, including parental depression, substance use, stress, and domestic violence \| Training; screening; response \| *SEEK* Study Questionnaire \| Not listed (urban, suburban, and rural) \| 2006-2009 \| 95 pediatricians and NPs \| Randomized controlled trial \| High (++++) \| 1. Knowledge: Knowledge scores on a health provider questionnaire did not differ between the treatment and control groups at 6, 18, or 36 months after the intervention (p>0.05). 2. Confidence/Self-Efficacy: Compared to those in the control group, providers in the intervention group reported greater improvement in comfort in addressing ACEs at 6 months (p<0.01), 18 months (p<0.01), and 36 months (p<0.05) after the intervention. 3. Screening Behavior: Providers in the intervention group improved screening rates by more than 20 percentage points for each screening of interest, a change that was not observed in the control group (p<0.001). 4. Clinical Care: Providers in the intervention group reported larger improvements in addressing household difficulties, including maternal depression, IPV, substance abuse, and stress, across all three time periods (6, 18, and 36 months) (p<0.05). \| \| Flanagan et al., 2018 (22) \| Adverse childhood experiences (omnibus) \| Training;  screening \| Behavioral Risk Factor Surveillance System (BRFSS); Connor-Davidson Resilience Scale (CD-RISC 10) \| Northern CA, USA \| 2016 \| 26 clinicians; 210 patients \| Prospective cohort, no comparison group; focus group discussions with providers \| Low (++) \| 1. Knowledge: After the program, providers' knowledge of ACEs increased from an average of 3.0 to 4.1 on a five-point Likert scale (p<0.001). Providers' knowledge of resources for patients with ACEs also increased from an average of 1.9 to 3.9 on a five-point Likert scale (p<0.001). 2. Confidence/Self-Efficacy: Providers' self-reported ability to inquire about ACEs increased from an average of 2.6 to 4.0 on five-point Likert scale (p<0.001). Self-reported ability to provide counsel on ACEs increased from 2.4 to 3.9 on a 5-point Likert scale (p<0.001). During focus groups, providers expressed that screening was easier than expected. 3. Referrals: Clinicians provided 64% of patients a referral or resources for ACE-related care. 4. Screening Behavior: Among eligible patients, a majority (78%) were screened. 88% of screened patients completed the screening questionnaire. During focus groups, providers shared that training, adequate workflows, and greater behavioral health resources increased willingness to conduct screenings. \| \| Gerlach et al., 2021 (73) \| Adverse childhood experiences (omnibus) \| Screening; response \| Not Reported \| Central TX, USA \| 2016 \| 114 providers \| Cross-sectional, survey \| Low (++) \| 1. Screening Behavior: 28.4% of providers did not screen for ACEs. Most commonly, providers (51.4%) screened for anyone in the home having a mental illness or substance use disorder. Least frequently screened ACEs were ethnic/racial discrimination (6.5%) and community violence (9.4%). 2. Clinical Care: The most common strategies to deal with ACEs were using on-site clinical staff and resources, including social workers or therapists (76.4%) or materials/publications (71.9%). In multivariate analysis, a one-unit increase in self-reported ACE knowledge increased the odds of implementing the strategies of identifying family strengths by 2.00 (p<0.05) and implementing other interventions by 1.83 (p<0.05). \| \| Glowa et al., 2016 (19) \| Adverse childhood experiences (omnibus) \| Screening \| CYW-ACEs Screening Tool \| ME, NH, and VT, USA (rural) \| 2015 \| 7 clinicians; 111 clinician surveys; 127 patients \| Prospective cohort, no comparison group \| Very low (+) \| 1. Knowledge: Clinicians reported that they learned new information by using the screening instrument (p<0.001). 2. Referrals: Use of screening did not result in a significant increase in referrals (p>0.05). 3. Screening Behavior: All 127 patients completed the ACEs questionnaire. None refused. 4. Clinical Care: Use of the screening rarely altered care delivery: It altered care 16.7% of the time among those with four or more ACEs and changed follow-up plans 1.4% of the time among all patients. 5. Other: 100% of clinicians reported that they felt the ACEs screener did not interfere with the visit. \| \| Kalmakis et al., 2018 (23) \| Adverse childhood experiences (omnibus) \| Response \| Adverse Childhood Experiences (ACE) Questionnaire \| MA, USA (rural) \| 2017 \| 71 adult patients \| Cross-sectional, survey; semi-structured interviews with patients \| Low (++) \| 1. Knowledge: After receiving training and screening two patients, providers indicated feeling "very confident" in their knowledge to screen for ACEs. 2. Confidence/Self-Efficacy: After receiving training and screening two patients, providers reported feeling "very comfortable" during the screening and “very confident” in their ability to screen. 3. Referrals: Providers referred 28% of the screened adults for follow-up care. \| \| van den Akker et al., 2001 (81) \| Traumatic events, including accidents, incest, and physical and sexual abuse \| Response \| Not Reported \| Netherlands \| Not listed \| 211 general practitioners \| Cross-sectional, survey; semi-structured interviews with providers \| Very low (+) \| 1. Knowledge: 20% of practitioners reported that they encounter barriers caring for children who are physically or sexually abused, with the most common barriers being lack of knowledge of signs of abuse (42%) and danger (48%), and insufficient skills to confront parents with suspected abuse (52%) or to start treatment (62%). \| | | | | | | | | | | |

| **C. Patient-Related Outcomes – Pediatric Patients** | | | | | | | | | | | | | |  |  |
| --- | --- | --- | --- | --- | --- | --- | --- | --- | --- | --- | --- | --- | --- | --- | --- |
| Authorship | Adversity Focus | Intervention Focus | Screening Instrument | Setting | Study Period | Analytic  Sample Size | | | Study Design | | Strength of Evidence | Patient-Related Outcomes | | |  |
| Bodendorfer et al., 2020 (65) | Adverse childhood experiences (omnibus) | Screening | Not Reported | Midwest USA | 2017-2018 | 13 primary care providers; 238 parents/guardians | | | Repeated cross-sectional, survey | | Low (++) | 1. Acceptability/Satisfaction: A majority of parents/guardians reported that the discussions on ACEs with their primary care provider was positive (76%) and comfortable (81%). | | |  |
| Carroll et al., 2005 (61) | Child abuse; maternal depression; family violence | Training; screening | Antenatal Psychosocial Health Assessment (ALPHA) | Ontario, Canada | Not listed | 48 providers; 227 patients | | | Randomized controlled trial | | Moderate (+++) | 1. Acceptability/Satisfaction: A majority of women were comfortable discussing items on the assessment form (72.7%) and thought it was part of their provider's job (76.3%). | | |  |
| Crenshaw et al., 2021 (67) | Adverse childhood experiences (omnibus) | Screening | Center for Youth & Wellness (CYW) ACEs Screening Tool | Not listed | 2018-2020 | 8 physicians; 232 families | | | Cross-sectional, survey | | Very low (+) | 1. Acceptability/Satisfaction: In interviews, caregivers reported several themes, including gratitude for conducing the screening and a desire to seek professional help to process these experiences. | | |  |
| Dubowitz et al., 2009 (86) | Child maltreatment | Training; screening; response | Safe Environment for Every Kid (*SEEK*) Study Questionnaire | Baltimore, MD, USA | 2002-2005 | 558 caregivers | | | Randomized controlled trial | | Moderate (+++) | 1. Change in Health Outcomes: Compared to those in the control group, caregivers in the intervention group reported fewer cases of severe or very severe physical assault (average weighted score of 0.11 vs. 0.33, p=0.04) and fewer cases of non-adherence to medical care (4.6% vs. 8.4%, p=0.05) and late immunizations (3.3% vs. 9.6%, p=0.002). 2. Other: Intervention families had fewer CPS reports than those in the control group (13.3% vs 19.2%, p=0.03). | | |  |
| Dubowitz et al., 2012 (85) | Child maltreatment | Training; screening; response | *SEEK* Study Questionnaire | Not listed | 2006-2009 | 1119 mothers | | | Randomized controlled trial | | High (++++) | 1. Change in Health Outcomes: One year after the intervention, mothers in the intervention group reported lower levels of psychological aggression (effect size=-0.12, p<0.05) and fewer cases of minor physical assault (effect size=-0.14, p<0.05) compared to mothers in the control group. | | |  |
| Eismann et al., 2019 (51) | Child maltreatment; parental depression; substance use; domestic violence; discipline; food insecurity | Training; screening; response | *SEEK* Study Questionnaire | Not listed | 2015-2016 | 16 providers; 1,190 targeted families | | | Cross-sectional, survey; semi-structured interviews with providers | | Low (++) | 1. Acceptability/Satisfaction: According to providers, caregivers were comfortable discussing ACEs. A small number of families declined to discuss ACEs. 2. Referrals: 14% of targeted families at the federally qualified health center, 25% at the family medicine practice, and 30% at the pediatric practice accepted referrals to specialty care services. | | |  |
| Eismann et al., 2023 (56) | Childhood adversity and toxic stress | Training; screening; response | *SEEK* Study Questionnaire | | Midwest, USA | | 2017-2019 | 27,419 pediatric patients | | Randomized controlled trial | | High (++++) | 1. Changes in Clinical Service: During the SEEK intervention, patients were significantly less likely to have a sick visit (OR=0.50), but were less likely of completing their well-child visit on time (OR=0.88). | | |
| Feigelman et al., 2011 (45) | Psychosocial risk factors, including parental depression, substance use, and stress; domestic violence; discipline; food insecurity | Training | *SEEK* Study Questionnaire | Not listed (urban) | Not listed | 95 residents; 558 families | | | Randomized controlled trial | | Moderate (+++) | 1. Acceptability/Satisfaction: Families seen by the intervention group of providers reported higher satisfaction with visits than those seen by control group providers (p<0.01). Six months after the intervention, however, these group differences were not sustained. | | |  |
| Garg et al., 2007 (58) | Psychosocial risk factors, including parental unemployment, lack of education, substance use, and depression; domestic violence; homelessness, and food insecurity | Training; screening; response | Well Childcare Visit, Evaluation, Community Resources, Advocacy, Referral, Education (WE CARE) Survey | | Not listed | | 2006 | 45 residents; 200 parents | | Randomized controlled trial | | High (++++) | 1. Acceptability/Satisfaction: Compared to those in the control group, intervention group parents had fewer unmet needs to discuss psychosocial topics (0.46 vs. 1.41, p=0.001). 2. Referrals: Compared to those in the control group, parents in the intervention group had a higher likelihood of contacting community resources for assistance (adjusted OR=17.3, p<0.001). | | |
| Kia-Keating et al., 2019 (26) | Adverse childhood experiences (omnibus); adverse community experiences, including discrimination, violence, and natural disaster | Screening | CYW-ACEs screening tool | Not listed | Not listed | 164 patients and caregivers | | | Cross-sectional, survey; semi-structured interviews with providers | | Very low (+) | 1. Referrals: Among parents eligible for and offered a referral, 77% agreed. Common reasons for declining included: lack of time, receiving services elsewhere, and relocating. | | |  |
| Marie-Mitchell et al., 2019 (27) | Child abuse and neglect; domestic violence; substance use; mental illness; incarceration | Screening | Whole Child Assessment (WCA) | San Bernardino, CA, USA | 2014-2017 | 1,100 patient medical charts; 30 caregivers | | | Repeated cross-sectional, non-survey; semi-structured interviews with providers | | Low (++) | 1. Acceptability/Satisfaction: 90% of parents/caregivers expressed no concerns with filling out the ACEs screener. Those who expressed concerns chiefly discussed discomfort disclosing financial and food insecurity. | | |  |
| Negriff et al., 2022 (89) | Adverse childhood experiences (omnibus) | Screening; response | Kaiser Permanente–Centers for Disease Control and Prevention (KP-CDC) Questionnaire with Adaptations from CYW-ACEs Screening Tool | Los Angeles County, CA | 2018-2021 | 4,030 children; 4,332 encounters | | | Repeated cross-sectional, non-survey | | Medium (+++) | 1. Referrals: Post-intervention children were 7.5 times (95% CI: .55-36.2, p<0.05) more likely to have a behavioral health visit if they screened positive for an ACE than compared to the pre-intervention period. This was sustained over time (interrupted time series). Adjusted rates were 4.33% preintervention to 32.48% postintervention. | | |  |
| Rosado et al., 2023 (57) | Adverse childhood experiences (omnibus) | Training; screening | ACEsPlus+ | | Florida | | Not listed | 2,347 children; 6 pediatricians | | Cross-sectional, survey; focus group discussions with providers | | Very low (+) | 1. Acceptability/Satisfaction: Parents reported feeling comfortable with questions from screeners. Parents were least comfortable talking about incarceration. Children were less comfortable with questions on family issues, incarceration, and sexual abuse (no percentages or significance tests reported). | | |
| Selvaraj et al., 2019 (29) | Adverse childhood experiences (omnibus); unmet needs, including parental unemployment, lack of education, and food and housing insecurity | Screening | Addressing Social Key (ASK) Questions for Health Questionnaire | Chicago, IL, USA | 2016-2017 | 2,569 parents or guardians | | | Cross-sectional, survey | | Low (++) | 1. Acceptability/Satisfaction: A majority of families (77%) felt comfortable filling out the Addressing Social Key (ASK) tool and 79% of families felt supported through the discussion with their provider. Most families (86%) thought that screening should continue. | | |  |
| Selvaraj et al., 2022 (78) | Adverse childhood experiences; family unmet social needs | Screening; response | Not Reported | | Chicago, IL | | 2018-2019 | 40 parents | | Semi-structured interviews with parents | | Very low (+) | 1. Acceptability/Satisfaction: Parents reported that they understood that ACEs were related to health, and that asking about ACEs means that health clinics can address ACEs. Parents did not reach consensus on their preferred method for addressing ACEs (i.e., with overall score, or with item-level scores) | | |
| Strait and Meagher, 2020 (87) | Adverse childhood experiences (omnibus) | Screening; response | CYW-ACEs Screening Tool | Not listed | Not listed | 12 patients | | | Qualitative | | Very low (+) | 1. Acceptability/Satisfaction: Most families and patients were open to discussing ACEs and its impact on toxic stress. | | |  |
| Woods-Jaeger et al., 2020 (90) | Adverse childhood experiences (omnibus); depression; social support | Response | Expanded ACEs Questionnaire | Midwest, USA | Not listed | 11 child caregivers | | | Prospective cohort, no comparison group | | Very low (+) | 1. Acceptability/Satisfaction: Caregivers generally reported all session content was helpful or very helpful and would recommend the program to others. 2. Other: Caregivers demonstrated consistent engagement with the intervention. They shared opinions and disclosed information during sessions and followed through with at-home activities and techniques. | | |  |

| **D. Patient-Related Outcomes – Adult Patients** | | | | | | | | | |  |
| --- | --- | --- | --- | --- | --- | --- | --- | --- | --- | --- |
| Authorship | Adversity Focus | Intervention Focus | Screening Instrument | Setting | Study Period | Analytic Sample Size | Study Design | Strength of Evidence | Patient-Related Outcomes | |
| Carroll et al., 2005 (61) | Child abuse; maternal depression; family violence | Training; screening | Antenatal Psychosocial Health Assessment (ALPHA) | Ontario, Canada | Not listed | 48 providers; 227 patients | Randomized controlled trial | Moderate (+++) | 1. Acceptability/Satisfaction: A majority of women were comfortable discussing items on the assessment form (72.7%) and thought it was part of their provider's job (76.3%). | |
| Flanagan et al., 2018 (22) | Adverse childhood experiences (omnibus) | Screening | Behavioral Risk Factor Surveillance System (BRFSS) | Northern CA, USA | 2016 | 26 clinicians; 480 eligible patients; 210 screened patients | Prospective cohort, no comparison group; focus group discussions with providers | Low (++) | 1. Acceptability/Satisfaction: A large majority of patients who completed the ACE screening and follow-up survey felt comfortable discussing ACEs with providers (93%) and filling out the screening questionnaire (91%). However, comfort was lower among those with a higher number of ACEs. 75% of those with no ACEs were "very comfortable" with the screening, compared to 60% of those with one to two ACEs (p=0.04) and 35% of those with three or more ACEs (p<0.001). About half (53%) reported that the screening increased trust in their provider. | |
| Gaba et al., 2022 (88) | Adverse childhood experiences | Screening | Pediatric ACEs and Related Life Events Screener (PEARLS) | USA (urban) | 2021 | 134 adult patients | Cross-sectional, survey | Very low (+) | 1. Acceptability/Satisfaction: There was no significant variation in patient-reported acceptability by race, ethnicity, gender, or age. 65% of participants reported ACE screening to be an acceptable part of primary care; however, over half of the small sample of Native American and Hispanic/Latinx patients did not consider screening to be acceptable. | |
| Glowa et al., 2016 (19) | Adverse childhood experiences (omnibus) | Screening | CYW-ACEs Screening Tool | ME, NH, and VT, USA (rural) | 2015 | 7 clinicians; 111 clinician surveys; 127 patients | Prospective cohort, no comparison group | Very low (+) | 1. Acceptability/Satisfaction: Clinicians reported on 98% of surveys that they felt the ACEs screener was acceptable to the patient. | |
| Goldstein et al, 2019 (77) | Adverse childhood experiences (omnibus) | Screening; response | CYW-ACEs Screening Tool; Primary Care PTSD Screen (PC-PTSD) | Milwaukee, WI, USA | 2021 | 40 adult patients | Prospective cohort, no comparison group | Low (++) | 1. Acceptability/Satisfaction: Patients reported high satisfaction with the intervention: 94% were “moderately satisfied” or “extremely satisfied”. 2. Referrals: Behavioral health referral acceptance rates were 39% one-month post-intervention and 31% at follow up two-months post-intervention. 3. Health Outcomes: Patients reported reduced rates of stress and health risk behaviors between baseline and post-intervention, including: self-reported perceived stress (p<0.001), unhealthy alcohol use (p=0.03), unhealthy nutrition habits (p=0.003), and risky sexual behaviors (p<0.001). At follow up, effects on perceived stress were sustained (p<0.001); no other effects were sustained (p>0.05). | |
| Woods-Jaeger et al., 2020 (90) | Adverse childhood experiences (omnibus); depression; social support | Response | Expanded ACEs Questionnaire | Midwest, USA | Not listed | 11 child caregivers | Prospective cohort, no comparison group | Very low (+) | 1. Acceptability/Satisfaction: Caregivers generally reported all session content was helpful or very helpful and would recommend the program to others. 2. Other: Caregivers demonstrated consistent engagement with the intervention. They shared opinions and disclosed information during sessions and followed through with at-home activities and techniques. | |

**Appendix 3. Quality Assessment of Evidence**

| Authors | Experi-  mental? | Longi-tudinal? | Multi-Site? | Comp. Group? | Sample Size? | Large Effect Size, Narrow CI? | Gradient or Consistent Results Pattern | Self-Report, or Proxy for Outcome of Interest? | Risk of Social Desirability Bias? | Risk of Selection Bias? |
| --- | --- | --- | --- | --- | --- | --- | --- | --- | --- | --- |
| Abatemarco et al., 2008 | No | Yes | Yes | No | Small (<100) | No | No | No | No | Yes |
| Abatemarco et al., 2018 | No | Yes | Yes | Yes | Small (<100) | No | Yes | Yes | Yes | Yes |
| Alhowaymel et al., 2023 | No | No | Yes | No | Medium (100-500) | No | No | Yes | Yes | Yes |
| Bannon et al., 2001 | No | Yes | Yes | No | Medium (100-500) | Yes | No | Yes | Yes | No |
| Berg-Poppe et al., 2022 | No | Yes | Yes | Yes | Small (<100) | No | No | Yes | Yes | Yes |
| Bodendorfer et al., 2020 | No | No | No | No | Medium (100-500) | No | No | Yes | Yes | Yes |
| Brennan et al., 2022 | No | Yes | Yes | No | Small (<100) | Yes | No | Yes | Yes | Yes |
| Bryant & van Graafeiland, 2020 | No | Yes | No | No | Small (<100) | Yes | No | Yes | Yes | Yes |
| Campbell et al., 2019 | No | No | Yes | No | Small (<100) | N/A | N/A | Yes | Yes | Yes |
| Candler et al., 2016 | No | No | Yes | No | Small (<100) | No | No | Yes | Yes | Yes |
| Carroll et al., 2005 | Yes | Yes | Yes | Yes | Medium (100-500) | Yes | No | No | No | No |
| Chokshi et al., 2020 | No | Yes | No | No | Small (<100) | Yes | No | Yes | Yes | No |
| Chung et al., 2019 | No | Yes | No | No | Large (>500) | No | Yes | No | No | No |
| Crenshaw et al., 2021 | No | Yes | No | No | Large (>500) | No | Yes | No | No | No |
| Cruz et al., 2023 | No | No | No | No | Small (<100) | No | No | Yes | Yes | Yes |
| Dara et al., 2013 | No | No | Yes | No | Medium (100-500) | Yes | Yes | Yes | Yes | Yes |
| DiGangi & Negriff, 2020 | No | No | Yes | No | Large (>500) | N/A | N/A | Yes | Yes | No |
| DiGiovanni et al., 2023 | No | Yes | Yes | No | Large (>500) | No | No | No | Yes | Yes |
| Dubowitz et al., 2009 | Yes | Yes | No | No | Large (>500) | No | Yes | Yes | Yes | Yes |
| Dubowitz et al., 2011 | Yes | Yes | Yes | Yes | Small (<100) | Yes | Yes | Yes | Yes | No |
| Dubowitz et al., 2012 | Yes | Yes | Yes | Yes | Large (>500) | Yes | Yes | No | No | No |
| Eismann et al., 2019 | No | No | Yes | No | Small (<100) | No | No | Yes | Yes | Yes |
| Eismann et al., 2023 | Yes | Yes | Yes | Yes | Large (>500) | Yes | No | No | No | No |
| Feigelman et al., 2011 | Yes | Yes | No | Yes | Large (>500) | Yes | Yes | Yes | Yes | No |
| Flanagan et al., 2018 | No | No | Yes | No | Medium (100-500) | No | No | Yes | Yes | Yes |
| Froula et al., 2017 | No | Yes | No | No | Small (<100) | No | Yes | Yes | Yes | No |
| Gaba et al., 2022 | No | No | No | No | Medium (100-500) | No | No | No | Yes | Yes |
| Garg et al., 2007 | Yes | Yes | No | Yes | Medium (100-500) | Yes | No | No | No | No |
| Gerlach et al., 2021 | No | No | Yes | No | Medium (100-500) | No | No | Yes | No | Yes |
| Glowa et al., 2016 | No | No | Yes | No | Medium (100-500) | Yes | No | Yes | Yes | Yes |
| Goldstein et al., 2019 | No | Yes | No | No | Small (<100) | Yes | Yes | Yes | Yes | Yes |
| Henry et al., 2003 | No | No | Yes | No | Medium (100-500) | Yes | Yes | Yes | Yes | Yes |
| Hoffman, et al, 2023 | No | No | No | No | Large (>500) | No | No | Yes | Yes | Yes |
| Hosdurga & Finlay, 2010 | No | No | Yes | No | Small (<100) | N/A | N/A | Yes | Yes | No |
| Jee et al., 2020 | No | Yes | No | No | Small (<100) | No | No | Yes | Yes | Yes |
| Kalmakis et al., 2018 | No | No | No | No | Small (<100) | No | No | Yes | Yes | Yes |
| Khan et al., 2005 | No | No | Yes | No | Medium (100-500) | No | No | Yes | No | No |
| Kia-Keating et al., 2019 | No | No | No | No | Medium (100-500) | No | No | Yes | Yes | No |
| Konijnendijk et al., 2019 | Yes | Yes | Yes | Yes | Small (<100) | No | No | No | No | No |
| Kuruppu et al., 2022 | No | No | Yes | No | Small (<100) | N/A | N/A | Yes | Yes | Yes |
| Lloyd et al., 2021 | No | Yes | No | No | Small (<100) | No | Yes | Yes | Yes | Yes |
| Liu et al., 2021 | No | No | No | No | Small (<100) | N/A | N/A | Yes | Yes | Yes |
| Marie-Mitchell et al., 2019 | No | Yes | No | No | Large (>500) | Yes | Yes | No | No | No |
| Marsicek et al., 2019 | No | Yes | No | No | Large (>500) | Yes | Yes | No | No | No |
| Negriff et al., 2022 | Yes | No | Yes | No | Large (>500) | No | Yes | No | No | No |
| Quizhpi et al., 2019 | No | Yes | No | No | Small (<100) | No | No | Yes | Yes | Yes |
| Popp et al., 2020 | No | No | Yes | No | Small (<100) | N/A | N/A | Yes | Yes | Yes |
| Reading et al., 2022 | No | No | No | No | Small (<100) | No | No | Yes | Yes | Yes |
| Rosado et al., 2023 | No | No | No | No | Small (<100) | No | No | Yes | Yes | No |
| Schmitz et al., 2019 | No | Yes | No | No | Small (<100) | No | Yes | Yes | Yes | Yes |
| Selvaraj et al., 2018 | No | No | Yes | No | Large (>500) | Yes | No | No | No | Yes |
| Selvaraj et al., 2022 | No | No | No | No | Small (<100) | No | No | Yes | Yes | Yes |
| Strait & Meagher, 2020 | No | No | No | No | Small (<100) | N/A | N/A | Yes | Yes | Yes |
| van den Akker et al., 2001 | No | No | No | No | Medium (100-500) | No | No | Yes | Yes | Yes |
| Walbeehm-Hol & Busari, 2022 | No | No | Yes | No | Large (>500) | No | No | Yes | Yes | Yes |
| Wilson et al., 2005 | No | Yes | No | Yes | Small (<100) | No | No | No | No | No |
| Woods-Jaeger et al., 2020 | No | No | No | No | Small (<100) | N/A | N/A | Yes | Yes | Yes |
| Yaun et al., 2022 | No | Yes | No | No | Medium (100-500) | N/A | No | No | No | No |
